# MUFFINN: cancer gene discovery via network analysis of somatic mutation data

**DOI:** 10.1186/s13059-016-0989-x

**Published:** 2016-06-23

**Authors:** Ara Cho, Jung Eun Shim, Eiru Kim, Fran Supek, Ben Lehner, Insuk Lee

**Affiliations:** Department of Biotechnology, College of Life Science and Biotechnology, Yonsei University, Seoul, Korea; EMBL-CRG Systems Biology Unit, Centre for Genomic Regulation (CRG), 08003 Barcelona, Spain; Universitat Pompeu Fabra (UPF), 08003 Barcelona, Spain; Division of Electronics, Rudjer Boskovic Institute, 10000 Zagreb, Croatia

**Keywords:** Cancer gene prediction, Cancer somatic mutation, Cancer genomes, Mutation frequency, Functional gene network, Pathway-centric analysis

## Abstract

A major challenge for distinguishing cancer-causing driver mutations from inconsequential passenger mutations is the long-tail of infrequently mutated genes in cancer genomes. Here, we present and evaluate a method for prioritizing cancer genes accounting not only for mutations in individual genes but also in their neighbors in functional networks, MUFFINN (MUtations For Functional Impact on Network Neighbors). This pathway-centric method shows high sensitivity compared with gene-centric analyses of mutation data. Notably, only a marginal decrease in performance is observed when using 10 % of TCGA patient samples, suggesting the method may potentiate cancer genome projects with small patient populations.

**Electronic supplementary material:**

The online version of this article (doi:10.1186/s13059-016-0989-x) contains supplementary material, which is available to authorized users.

## Background

Cancer is a complex genetic disease caused by DNA abnormalities [1]. For this reason, substantial genetic and genomic efforts have been undertaken to identify causal cancer genes. Advanced DNA sequencing technologies have accelerated the discovery of cancer genes by cataloging the genetic aberrations in cancerous cells [2, 3] and consortia such as The Cancer Genome Atlas (TCGA) [4] and the International Cancer Genome Consortium (ICGC) [5] have undertaken the systematic profiling of genomic alterations in many cancer types.

A major challenge in cancer gene discovery via somatic mutation profiling is the driver and passenger problem [6]. A considerable number of somatic mutations identified by sequencing are passenger mutations with no impact on cancer progression. In contrast, a relatively small number of driver mutations that confer a selective growth advantage are expected in each sample [1, 7]. Distinguishing driver from passenger mutations is critical to reduce false positives in sequencing-driven cancer gene discovery.

The most intuitive and commonly used approach for distinguishing drivers from passengers are frequency-based methods that quantify the significance of the mutation frequency of each gene or region compared with a background mutation rate (BMR), which varies substantially across the genome and for different sequence contexts [8]. In frequency-based methods, genes that are mutated at higher rates than expected are declared as cancer driver genes. However, estimating an accurate BMR, which is the key step of the frequency-based methods, is not a trivial task. To take into account the wide dynamic range of the BMR, more sophisticated methods were suggested, such as MutSigCV [9], InVEx [10], and MuSiC [11]. They use elaborate methods for BMR estimation across patients, chromosomal locations, and mutational spectra.

Other approaches for distinguishing drivers from passengers consider the predicted functional impact of mutations on a protein’s activity. SIFT (Sorting Intolerant From Tolerant) [12] and PolyPhen-2 (Polymorphism Phenotyping v2) [13] are two commonly used methods for assessing the functional impact of protein variants, but they are not specialized for cancer gene prediction. Therefore, MutationAssessor [14] and CHASM (Cancer-specific High-throughput Annotation of Somatic Mutations) [15] were developed specifically for the assessment of the functional impact of variants in cancer. Other methods include TransFIC (TRANSformed Functional Impact for Cancer) [16], which considers the basal tolerance to germline single nucleotide variants, and CONDEL [17], which integrates multiple methods.

Despite significant progress in reducing false positives during the past several years, mutation-based cancer gene prediction is still underpowered, suffering from low sensitivity because of the phenomenon of the long-tail of infrequently mutated genes. Whereas frequency-based methods can identify driver genes amongst the genes that are frequently mutated in patients, they are ineffective in identifying drivers amongst infrequently or rarely mutated genes [18]. To obtain sufficient statistical power to detect cancer driver genes with low mutation frequency, a very large population of cancer patients would have to be sequenced [19]. Similarly, because of both high false positive and false negative rates, methods assessing the functional impact of mutations also have a limited capability to identify drivers amongst infrequently mutated genes [7].

An important observation from analyses of the landscape of cancer mutation genomes performed to date has been the convergence of individual mutations into cellular pathways [18]. Although somatic mutations in different genes are observed in different patients, these mutated genes tend to fall into a limited number of recurrently mutated pathways and processes in any particular type of cancer. This supports the hypothesis that cancer is a disease of pathway defects and has stimulated the development of pathway- or network-centric approaches for analyzing cancer somatic mutation data. For example, mutated genes in cancer genomes can be prioritized by their network connections to other mutated genes or known cancer genes [20]. DriverNet [21] and OncoIMPACT [22] prioritize mutated genes based on connections to dysregulated genes in cancer using matched expression data. ReMIC [23], VarWalker [24], and HotNet2 [25] identify cancer modules which comprise driver genes by diffusing mutation information throughout a network. These methods, however, either require extra information such as known cancer genes and matched expression data or focus on the discovery of cancer modules rather than prioritizing individual genes as cancer drivers.

Here we present a cancer gene prioritization method based on a pathway-centric analysis of mutation data, MUFFINN (MUtations For Functional Impact on Network Neighbors), that integrates mutational information for individual genes and their neighbors in co-functional networks. MUFFINN is highly predictive for known cancer genes, particularly for genes with low mutation occurrence among cancer patients, with the identification of drivers amongst these genes having substantially higher sensitivity than conventional methods based on gene-centric analysis of mutation data. MUFFINN works effectively with both pan-cancer and individual cancer type samples. MUFFINN has only marginally reduced predictive performance when using only 10 % of TCGA patient samples, suggesting that it will be a valuable method for small-scale cancer genome projects and in the initial stages of larger projects. Using mutation frequency data for 18 types of cancers from TCGA (as of August 2014), we identified approximately 200 novel candidates for cancer genes that were not successfully prioritized by conventional gene-centric methods such as MutSig2.0, MutSigCV, and MutationAssessor. We were able to find supporting evidence for many of them being bona fide drivers. Furthermore, we provide a companion web-based prediction server (http://www.inetbio.org/muffinn), which allows researchers to prioritize candidate cancer genes by submitting mutation occurrence data.

## Results

### Overview of MUFFINN

**Fig. 1:**
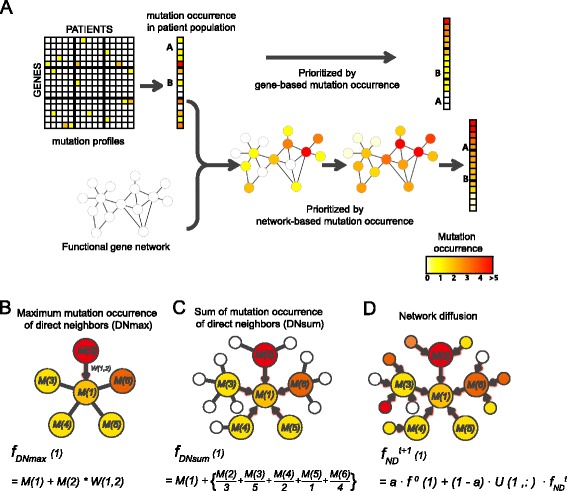
Overview of MUFFINN. **a** While conventional methods based on mutation data prioritize genes with mutation frequency only, MUFFINN prioritizes genes based on the mutation frequency of each gene as well as that of its neighbors in a functional network. Various network algorithms incorporate mutational information of network neighbors: **b** mutation frequency of direct neighbor genes of maximum mutation frequency; **c** mutation frequency of all direct neighbors with normalization by the number of their network neighbors; and **d** mutational frequency of all genes of the entire network with diffused weight through the network

From the observed clustering of genes somatically mutated in cancers into pathways [18], we hypothesized that a gene is more likely to represent a true cancer driver if it is functionally associated with other genes mutated in cancer. Therefore, we devised a method that considers the mutation information of both a given gene and its neighbors in a functional network. Unlike conventional cancer gene classifiers based only on the mutation information of individual genes, our method, MUFFINN, accounts for both the mutation frequency of each gene as well as those of its network neighbors (Fig. 1a). If a gene with low probability of being involved in cancer due to its low mutation frequency has many mutations among its network neighbors, MUFFINN will reprioritize it as a highly probable candidate.

For network-based mutation data analysis, we may consider mutations only in the direct neighbors of a gene or those of the entire network. Two ways to take into account mutational information among direct neighbors are to either consider mutations in the most frequently mutated neighbor (direct neighbor max, DNmax; Fig. 1b) or to consider mutations in all direct neighbors with normalization by their degree connectivity (direct neighbor sum, DNsum; Fig. 1c). We also hypothesized that network-based prediction might also be improved by taking into account indirect neighbors using diffusion algorithms [26]. We therefore tested MUFFINN with various network diffusion algorithms (Fig. 1d).

### MUFFINN is highly predictive for known cancer genes

For the analysis of MUFFINN in cancer gene prediction, we employed somatic mutation data for each cancer type from TCGA and two independently developed functional gene networks, HumanNet [27] and STRING v10 [28]. Both networks consist of interactions between genes predicted to share biological functions. To assess the predictive power of classifiers for cancer genes, we ideally need an accurate, comprehensive, and unbiased gold-standard cancer gene set. Unfortunately, such a cancer gene set is not available so we generated five distinct gold-standard cancer gene sets from various sources of annotations: (i) 422 cancer genes from the Cancer Genome Census database (CGC) [29] as of October 2012; (ii) a CGC subset of 118 cancer genes which act in cancer via point mutations (CGCpointMut); (iii) 124 cancer genes based on the characteristic mutational patterns for oncogenes and tumor suppressor genes (20/20 rule) [1]; (iv) 288 high-confidence driver genes based on a rule-based approach (HCD) [30]; and (v) 797 human orthologs of mouse cancer genes identified by insertional mutagenesis (MouseMut) [31, 32]. Each gold-standard cancer gene set has a different trade-off between accuracy, comprehensiveness, and bias. For instance, cancer genes annotated by CGC are regarded as highly accurate at the expense of high bias toward translocations in blood cancer, while the largest set of 797 cancer genes identified by mutagenesis in mice is comprehensive yet not extensively validated. Although each cancer gene set is biased towards particular features or study methods, consistently high ranking of a classifier across the five cancer gene sets would be sufficient evidence of its predictive performance. To evaluate the effectiveness of the pathway-centric approach, we compare the performance of MUFFINN with the performance of three popular methods based on gene-centric analyses of somatic mutation data: MutSig2.0, MutSigCV [9], and MutationAssessor [14].

**Fig. 2:**
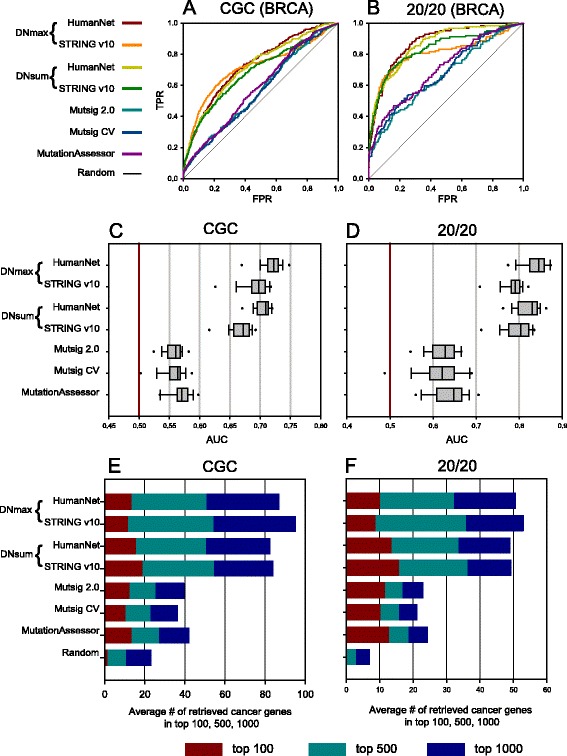
Assessment of predictive power of MUFFINN for cancer genes. ROC analyses on prediction of cancer genes annotated by CGC (**a**) and the 20/20 rule (**b**) were performed for MUFFINN with various combinations of two network algorithms using direct neighbors (DNmax and DNsum) and two networks (HumanNet and STRING v10) and conventional frequency-based methods, MutSig 2.0, MutsigCV, and MutationAssessor, with mutation data derived from breast cancer type (BRCA). The same analysis was repeated for all 18 cancer type samples and the results are summarized as the distribution of 18 AUC scores for cancer genes annotated by **c** CGC and **d** the 20/20 rule. Prediction powers for top candidates were assessed by cumulative numbers of retrieved cancer genes annotated by **e** CGC and **f** the 20/20 rule within the top 100, 500, and 1000 with the same analysis setting. Results from all the assessment tests indicate the generally improved performance of MUFFINN over the tested gene-centric cancer gene classifiers. *FPR* false positive rate, *TPR* true positive rate

To assess the predictive performance for the gold-standard cancer genes, ROC (receiver operating characteristic) analysis was performed for each type of cancer. We first tested MUFFINN by using mutation occurrence data only in direct neighbors (NDmax and NDsum) and found a generally higher predictive performance than gene-by-gene analyses. For example. MUFFINN showed higher performance in predicting cancer genes annotated by CGC [29] and the 20/20 rule [1] using mutation data for breast cancer with either HumanNet or STRING v10 (Fig. 2a, b). The ROC analysis results can be summarized into area under the ROC curve (AUC) scores. Thus, we used the distribution of AUC scores across 18 cancer types to summarize the general prediction performance across different cancer types with different combinations between network algorithms (DNmax and DNsum) and functional networks (HumanNet and STRING v10). For all five gold-standard cancer gene sets, MUFFINN consistently outperforms all three gene-centric methods that do not account for mutation frequency of network neighbors (Fig. 2c, d; Additional file 1: Figure S1a–c).

For practical reasons, in cancer gene discovery only the top-ranked candidate genes might enter into the follow-up experimental validation. Hence, the prediction power for the top ranked candidates is likely a more relevant metric for the real application of cancer gene classifiers. We previously demonstrated that high performance of a network-based gene prioritizer based on all genes cannot guarantee successful prioritization for the top ranked candidate genes for phenotypes including human diseases [33]. Therefore, we also assessed the predictive power for the top-ranked candidates based on the average number of retrieved gold-standard cancer genes across 18 cancer types for the top 100, 500, and 1000 candidates. We observed similar prediction power among all MUFFINN classifiers and gene-centric classifiers for the top 100 candidates. However, all MUFFINN classifiers showed substantially higher predictive power for the top 500 and 1000 candidates based on all five gold-standard cancer gene sets (Fig. 2e, f; Additional file 1: Figure S1d–f). These results indicate that MUFFINN not only achieved a specificity for top-tier candidates as high as current state-of-the-art gene-centric algorithms but also provides substantially more opportunities for the discovery of novel cancer genes by maintaining high sensitivity for extended ranges of ranked candidates.

To test the robustness of MUFFINN to network coverage, we repeated the predictions using the top 75, 50, and 25 % of network links. We observed no significant loss in retrieval rate for all five gold-standard cancer gene sets using the smaller networks (Additional file 1: Figures S2 and S3).

### Considering mutations in indirect neighbors does not improve predictive performance

MUFFINN can also use mutations in indirect neighbors by diffusing mutation occurrence information throughout the network (Fig. 1d). Recently, diffusing information through a network has proven useful in various network-based gene prioritizations [26]. Therefore, we tested MUFFINN with three popular network diffusion algorithms for cancer gene predictions: (i) Gaussian smoothing (GS); (ii) random walk with restart (RWR); and (iii) iterative ranking (IR), which has been popularized as the PageRank algorithm of internet search engines. The three algorithms diffuse initial node information, here mutational occurrences, to all the genes of the network. Consequently, not only direct neighbors but also all the genes of the network can affect the probability of a gene being a cancer driver with different weights.

**Fig. 3:**
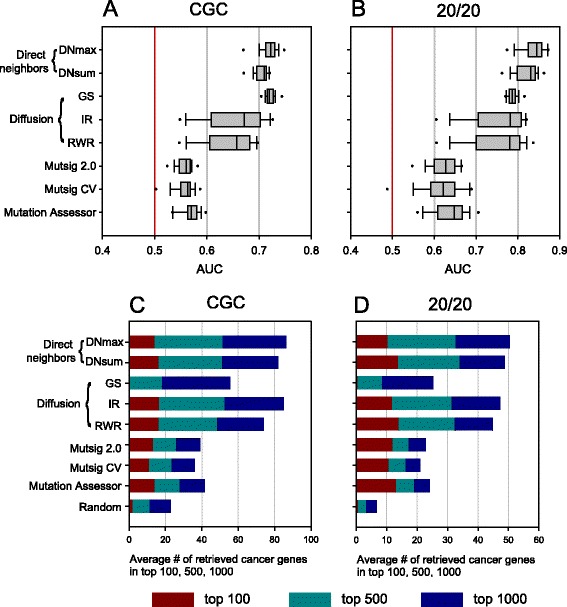
MUFFINN performs best by using mutation information of direct neighbors only. Performance assessment was conducted similarly to those of Fig. 2 for different network algorithms, including three diffusion algorithms on HumanNet: distributions of 18 AUC scores for cancer genes annotated by **a** CGC and **b** the 20/20 rule, and cumulative numbers of retrieved cancer genes annotated by **c** CGC and **d** the 20/20 rule within the top 100, 500, and 1000. MUFFINN shows higher predictive power for cancer genes by using mutation information of direct neighbors only than by using all genes of HumanNet with various network diffusion algorithms

Interestingly, we did not observe any improvement in predicting the cancer genes of all five gold-standard sets using the mutation occurrence data of indirect neighbors. Indeed, predictive power for all genes based on AUC scores across 18 cancer types in general decreases in MUFFINN with the network diffusion algorithms (Fig. 3a, b; Additional file 1: Figure S4a–c). Predictive performances for the top 100, 500, or 1000 candidates were comparable, with the exception of GS, but did not show significant improvement by using diffused mutational data from indirect neighbors in the network (Fig. 3c, d; Additional file 1: Figure S4d–f). From these results we conclude that network-based mutation analysis for cancer gene prediction needs mutation data of direct neighbors only.

We observed comparable performance between two alternative methods based on direct neighbors, DNmax and DNsum, across 18 cancer types. The different effectiveness of the two methods among cancer types could be attributed to differences in mutation distribution among member genes of cancer pathways. If mutations are evenly spread among members of cancer-related pathways for the given cancer type, DNsum works more effectively. Conversely, if mutations concentrate on a few hubs of cancer-related pathways for the given cancer type, adding more importance to the mutational information of the hub, DNmax, could be more effective for identifying additional cancer genes. We tested whether integration of the two distinct network algorithms, DNsum and DNmax, improves prediction power when using either the higher probability or the joint probability of the two classifiers. However, none of the integrated classifiers showed a significant improvement (data not shown). Therefore, we advise using both network algorithms and choosing the better performing classifier for given input data based on an evaluation using known cancer genes amongst the top ranked candidates.

### MUFFINN is predictive for cancer genes with only dozens of sequenced samples

**Fig. 4:**
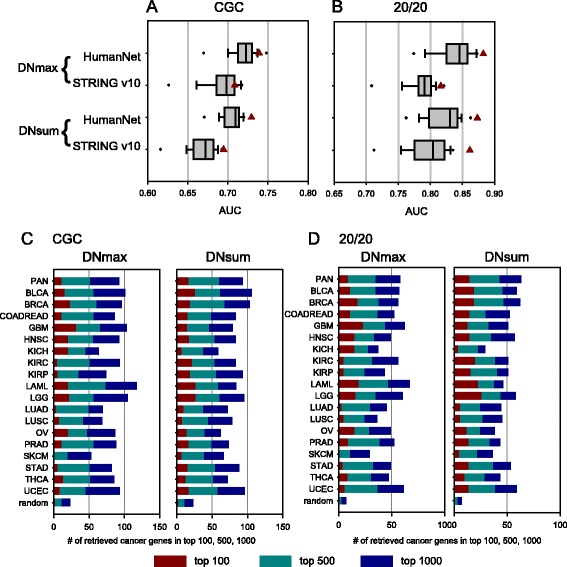
Comparison of MUFFINN predictive power with different cancer mutation data for samples from 18 types of cancer and pan-cancer samples. Distribution of AUC of MUFFINN using direct neighbors on HumanNet for cancer genes annotated by **a** CGC and **b** the 20/20 rule with pan-cancer samples and samples for 18 types of cancer. The AUC for the pan-cancer data is indicated by *red triangles* and those for the 18 types of cancer are represented as *box plots*. The number of retrieved cancer genes annotated by **c** CGC and **d** the 20/20 rule in the top 100, 500, and 1000 candidates by MUFFINN with pan-cancer samples and samples for 18 types of cancer. *BLCA* urothelial bladder cancer, *BRCA* breast cancer, *COADREAD* colon and rectal adenocarcinoma, *GBM* glioblastoma multiforme, *HNSC* head and neck squamous cell carcinoma, *KICH* chromophobe renal cell carcinoma, *KIRC* clear cell kidney carcinoma, *KIRP* papillary kidney carcinoma, *LAML* acute myeloid leukemia, *LGG* lower grade glioma, *LUAD* lung adenocarcinoma, *LUSC* lung squamous cell carcinoma, *OV* ovarian serous cystadenocarcinoma, *PRAD* prostate adenocarcinoma, *SKCM* cutaneous melanoma, *STAD* stomach adenocarcinoma, *THCA* papillary thyroid carcinoma, *UCEC* uterine corpus endometrial carcinoma

Pan-cancer data have been suggested to have merit over data sets for individual cancer types in cancer gene discovery because the larger number of samples increases statistical power [34]. Although MUFFINN effectively predicted cancer genes using mutation data derived from individual types, we also tested to what extent the collective power of pan-cancer data can improve predictions. Interestingly, we observed only a marginal improvement in prioritizing cancer genes among all human genes using pan-cancer data (Fig. 4a, b; Additional file 1: Figure S5a–c). Notably, mutation data for some types even outperformed the pan-cancer data in prediction of known cancer genes within the top 100, 500, or 1000 candidates (Fig. 4c, d; Additional file 1: Figure S5d–f).

Based on the low dependence on the abundance of mutation data observed from the analysis with pan-cancer data, we hypothesized that MUFFINN could be an effective cancer gene classifier when data are available for only dozens of sequenced samples. Therefore, we tested whether MUFFINN can predict cancer genes effectively with only 10 % of randomly selected samples from the TCGA database, which falls into the range of 6–98 samples for each type of cancer (chromophobe renal cell carcinoma (KICH) and breast cancer (BRCA) included 66 and 987 patients, respectively, in the TCGA database we used).

**Fig. 5:**
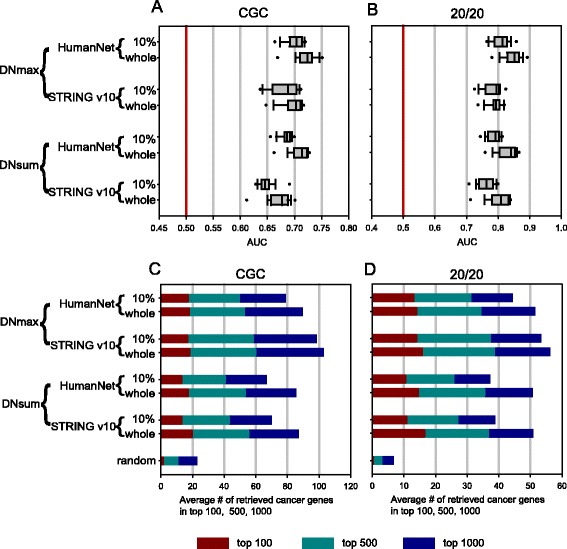
MUFFINN effectively predicts cancer genes with only 10 % of TCGA samples. Comparison of MUFFINN predictions for cancer genes annotated by **a** CGC and **b** the 20/20 rule between using all cancer samples and using only 10 % of the cancer samples. AUC scores for 18 cancer types when using all samples and when using 10 % of samples are represented as *box plots*. Performance comparisons were also conducted based on the number of retrieved cancer genes annotated by **c** CGC and **d** the 20/20 rule in the top 100, 500, and 1000 candidates. Notice that the scores for the 10 % of samples are all based on the average of 100 random samples. In general, MUFFINN shows only a marginal decrease in performance when using 10 % of samples compared with all samples

We assessed the predictive power for cancer genes using 100 random samples comprising 10 % of the original cancer samples and the distribution of AUC scores. Notably, we observed only a marginal decrease in prediction power, particularly for the top ranked candidates (Fig. 5; Additional file 1: Figure S6). These results illustrate how MUFFINN can overcome the long-tail phenomenon of cancer mutation data in cancer gene prediction. With only dozens of patients, infrequently mutated genes in the long tail are not likely to be identified as mutated genes. However, the frequently mutated genes are located within cancer pathways and propagate information via the network to other members of the pathway for which no mutations had yet been identified. Mutations in these genes will likely be identified in the future as more patients are sequenced and the sample size increases.

### Accounting for mutational heterogeneity is not important for MUFFINN

**Fig. 6:**
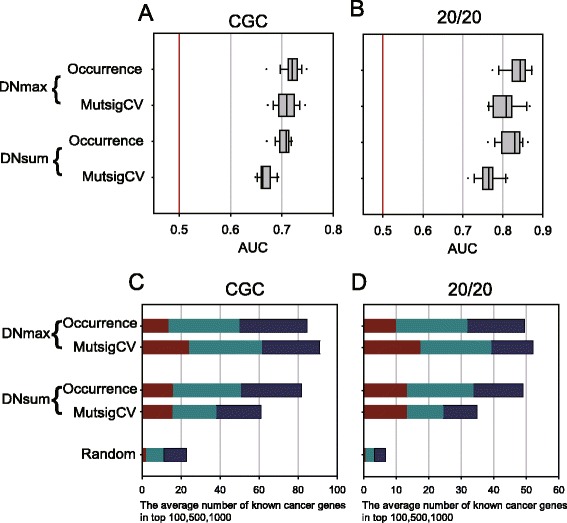
MUFFINN is not significantly improved by using MutsigCV scores. MUFFINN was performed with raw mutation frequencies or MutsigCV scores, which are normalized by BMR and mutational heterogeneity. HumanNet was used as the network model. Comparison of predictions for cancer genes annotated by **a** CGC and **b** the 20/20 rule. AUC scores for 17 cancer types (LAML was excluded in the analysis due to the indiscriminative MutsigCV scores due to the low mutation rate in leukemia) are represented as *box plots*. Performance comparisons were also conducted based on the number of retrieved cancer genes annotated by **c** CGC and **d** the 20/20 rule in the top 100, 500, and 1000 candidates

The major source of false positive cancer driver genes in frequency-based analyses of somatic mutation data is mutational heterogeneity due to mutation signature biases, gene expression levels, and DNA replication time/chromatin organization [8]. Normalization of observed mutation frequencies by gene-specific background mutation rates incorporating expression level, replication time, and patient-specific mutation frequencies, as implemented by the MutsigCV method, can eliminate most of the spurious candidate driver genes [9]. To test if correction of mutation frequencies by those factors can also improve our network-based cancer driver gene predictions, we compared MUFFINN predictions for the five gold-standard cancer gene sets when using raw mutation frequencies and MutsigCV scores. Note that acute myeloid leukemia (LAML) was excluded from this analysis because all genes in LAML have indiscriminative MutsigCV scores due to the low mutation rate in leukemia. We observed generally higher prediction powers for all five gold-standard cancer gene sets among 17 types when using raw mutation frequencies than when using MutsigCV scores (Fig. 6a, b; Additional file 1: Figure S7a–c), except for a slight improvement using MutsigCV with NDmax for the top 100 candidates (Fig. 6c, d; Additional file 1: Figure S7d–f). Elimination of candidate genes with high BMR seems effective in network-based prediction based on a single gene of maximum mutation occurrence among neighbors (i.e., DNmax). However, the normalizing effect of accounting for mutational heterogeneity is much reduced for the network-based prediction using mutations of all neighboring genes. These results indicate that accounting for mutational heterogeneity has, in some respects, a similar effect as taking into account pathway-level mutational burden. For gene-centric prediction algorithms, normalizing the mutation occurrence of each gene by transcription level, replication timing, and other such factors that affect mutation prevalence successfully filters out many false positives. MUFFINN, however, uses the information of group-wise mutational burden, enabling it to be more resistant to the influence of genes with intrinsically high mutation rates.

### MUFFINN predicts cancer genes not identifiable by gene-centric mutation analysis

Next we tested whether MUFFINN, which conducts pathway-centric analysis of mutation data, can predict cancer genes that are not identifiable by a gene-by-gene analysis of mutation data. To focus our validation on candidates only predicted by MUFFINN, we collected genes ranked in each cancer type within the top 1000 by MUFFINN with HumanNet but not by all three gene-centric methods, MutSig2.0, MutSigCV, and MutationAssessor, from 18 cancer types. We then excluded annotated cancer genes by CGC or the 20/20 rule from the collected candidates, resulting in 199 novel cancer genes. We then carried out a comprehensive literature review for the 199 candidate genes and found some level of association with cancer for 128 genes (~64 %), as summarized in Additional file 1: Table S1.

**Fig. 7:**
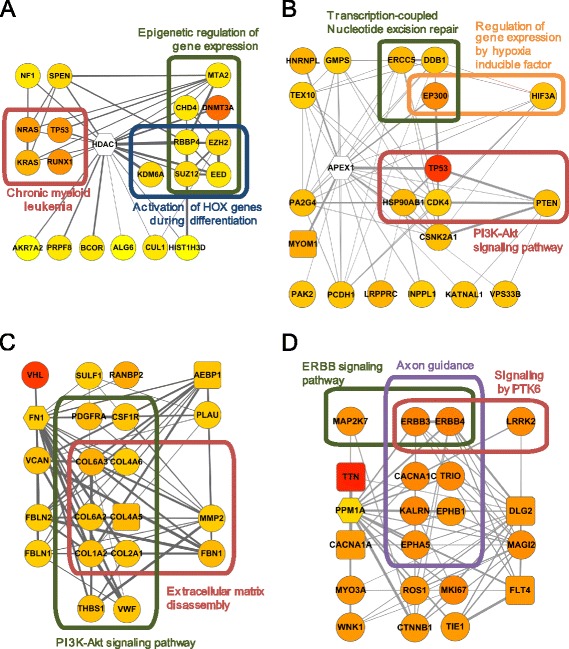
Networks of candidate cancer genes predicted by MUFFINN and their direct neighbors on HumanNet. Networks are visualized using Cytoscape for **a** HDAC1, **b** APEX1, **c** FN1, and **d** PPM1A. *Edge thickness* represents edge weight (likelihood of two genes being involved in the same pathway) by HumanNet and *node color scale* represents the mutational frequency of each gene, where higher mutation frequency is indicated as more reddish color. Genes with no mutation occurrence at all are indicated as an *empty node*. The subjected candidate genes, their neighboring candidate genes, and other genes are represented as *hexagonal*, *rectangular*, and *circular nodes*, respectively. Genes for each enriched pathway are grouped by a curved box

We assigned the 199 candidate genes into one of five classes. Figure 7 illustrates networks for representative candidates and the top 20 network neighbors of each for four classes, class 1 through 4, whose association with cancer was supported by the literature survey. To investigate cancer-related pathways among the network neighbors, we performed an enrichment analysis for three pathway annotations, KEGG pathway [35], Reactome pathway [36], and Gene Ontology (GO) biological process [37], using Fisher’s exact test. Class 1 includes 11 genes that are already reported as cancer genes but not annotated by CGC or the 20/20 rule dataset. HDAC1, ranked 29th by MUFFINN yet below 6000th by all three gene-centric methods in LAML samples, is a deacetylase, a chromatin modifying enzyme, and has been reported to be involved in myeloid leukemia cell proliferation [38]. Agreeing with the known functions, pathway annotations such as “epigenetic regulation of gene expression” (Reactome pathway, *P* = 2.87e-13), and “chronic myeloid leukemia” (KEGG pathway, *P* = 1.61e-08) are enriched among HDAC1 network neighbors (Fig. 7a). HDAC1 neighbor genes are also enriched for “activation of HOX genes during differentiation” (Reactome pathway, *P* = 3.70e-08), which is known to be involved in oncogenesis [39]. PLK1 is also known as an oncoprotein in leukemia [40] and ranked by MUFFINN (42nd) yet not by three gene-centric methods (below 9000th) in LAML samples. FLT4 (18th by MUFFINN yet below 4000th by all three gene-centric methods in LAML samples), ATR (28th by MUFFINN yet below 10,000th by all three gene-centric methods in BRCA samples) and MAP2K2 (fifth by MUFFINN yet below 11,000th by all three gene-centric methods in papillary thyroid carcinoma (THCA) samples) were recently added to the updated CGC gene list.

Class 2 includes 14 genes known to increase cancer susceptibility through germline variants. For example, HIF1A (16th by MUFFINN yet below 8000th by all three gene-centric methods in BRCA samples) polymorphism contributes to the risk of gastrointestinal cancer [41] and modulates the response to chemotherapy after surgery in patients with colorectal cancer [42]. Germline nucleotide variants in OBSCN (fifth by MUFFINN yet below 6000th by the gene-centric methods in papillary kidney carcinoma (KIRP) samples) were found in highly aggressive tumors such as glioblastoma, melanoma, and pancreatic carcinoma [43] and involved in cancer predispositions. Likewise, germline variants in APEX1 (14th by MUFFINN yet below 13,000th by the gene-centric methods in head and neck squamous cell carcinoma (HNSC) samples) is known to increase the risk of breast cancer development by contributing apurinic/apyrimidinic (AP) site accumulation in DNA [44]. Indeed, the genes functionally associated with APEX1 are enriched for a relevant pathway, “transcription-coupled nucleotide excision repair” (GO biological process, *P* = 7.45e-05; Fig. 7b). Essential roles for mammalian AP endonuclease in telomere maintenance have been reported [45], which supports the association of APEX1 with cancer development. We also found enrichment of APEX1 neighbors for “PI3K-Akt signaling pathway” (KEGG pathway, *P* = 5.01e-04) and “regulation of gene expression by hypoxia-inducible factor” (Reactome pathway, *P* = 5.49e-05), both of which are well known cancer therapeutic target signaling pathways [46, 47].

Class 3 includes 14 genes known to be involved in cancer by copy number variation (CNV) or structural variation (SV). For example, deletion of PTP4A3 (49th by MUFFINN yet below 10,000th by the gene-centric methods in KIRP samples) reduces the tumor-initiation ability in cancer [48] and PPAPDC1B (45th by MUFFINN yet below 16,000th by the gene-centric methods in colon and rectal adenocarcinoma (COADREAD) samples) is suggested to be a common driver in the 8p11-12 amplicon in breast, pancreatic, and lung cancer [49]. FN1 (15th by MUFFINN yet below 8000th by the gene-centric methods in clear cell kidney carcinoma (KIRC) samples) is a novel fusion partner of ALK in myofibroblastic tumors [50]. FN1 encodes fibronectin 1, an extracellular matrix component, and the network neighbors of FN1 were found to be enriched for “extracellular matrix disassembly” (GO biological process, *P* = 2.78e-15; Fig. 1c). The extracellular matrix has been recently reported to modulate the hallmarks of cancers [51]. In addition, FN1 network neighbors are enriched for a well-known cancer signaling pathway, the PI3K-Akt signaling pathway (KEGG pathway, *P* = 1.81e-14) [46].

Class 4, to which we assigned a total of 89 genes, is associated with cancer via expression regulation. For example, ACTN1 (33rd by MUFFINN yet below 8000th by the gene-centric methods in uterine corpus endometrial carcinoma (UCEC) samples) is known to have a tumor-specific splice variant in many types of cancer [52]. DCC (32nd by MUFFINN yet below 7000th by the gene-centric methods in HNSC samples), a putative candidate tumor suppressor, is inactivated by promoter hypermethylation in head and neck cancer [53] and loss of PPM1A (23rd by MUFFINN yet below 5000th by the gene-centric methods in stomach adenocarcinoma (STAD) samples) expression enhances invasion and epithelial-to-mesenchymal transition in bladder cancer [54]. Interestingly, genes functionally associated with PPM1A turned out to be enriched for “axon guidance” (GO biological process, *P* = 4.62e-07; Fig. 7d) and PPM1 has been reported as a regulator for axon termination and synapse formation in *Caenorhabditis elegans* [55]. Because many axon guidance molecules are also involved in regulation of cell migration and apoptosis [56], the enrichment of axon guidance genes among network neighbors may be informative for the association of PPM1A with cancer. We also found other cancer-associated signaling pathways enriched, such as “signaling by PTK6” [57] (Reactome pathway, *P* = 5.26e-05) and “ErbB signaling pathway” [58] (KEGG pathway, *P* = 1.26e-04) among PPM1A neighbors. ING1 (30th by MUFFINN yet 11,000th by the gene-centric methods in BRCA samples) was recently reported as a validated target of microRNA let-7b, which suppresses gastric cancer malignancy [59], and its down-regulation in breast cancer promotes metastasis [60]. Interestingly, many of the recent studies suggested a relationship between cancer and a candidate, DROSHA (61st by MUFFINN yet below 18,000th by the gene-centric methods in BRCA samples), which is involved in microRNA processing, through prognostic values [61], expression changes in breast cancer [62], and genetic variations [63].

As described above, MUFFINN predicted many cancer genes that have been missed by annotators or are infrequently mutated and yet have been previously implicated as cancer genes by germline variation, CNV, SV, or expression regulation. Cancer risk is affected in many ways other than just somatic mutations of coding sequences. Interestingly, TTN was top ranked by MUFFINN because its network neighbors also have many somatic mutations. TTN has been excluded from candidates in many predictions by frequency-based methods because its particularly high mutation frequency could be attributed to its large gene size. However, a recent study demonstrated that some network modules which confer significance in cancer subtyping are enriched for long genes such as TTN, which suggests that long genes should not necessarily be ignored by default in cancer gene studies [64].

Class 5 includes 71 candidate genes for which we were not able to find any additional evidence for association with cancer in published studies to date. These candidates are completely novel and need to be subject to further investigation for their association with human cancer in the future.

### Comparison between MUFFINN and HotNet2

Recently, HotNet2, a state-of-the-art software for identifying cancer driver genes by diffusing mutational burden through protein–protein interaction networks, has been applied to TCGA pan-cancer data and identified 144 candidates for cancer genes [25]. Although both methods are based on analyzing the network distribution of cancer somatic mutations, MUFFINN has several technical advantages over HotNet2: (i) MUFFINN prediction can be conducted via a web server (http://www.inetbio.org/muffinn/); (ii) MUFFINN runs much faster because an iterative network search is not necessary for the best performing DNsum and DNmax algorithms; (iii) MUFFINN provides probability scores for all candidate genes. To compare the performance of the two network-based cancer gene prediction methods, we reran MUFFINN on the TCGA pan-cancer somatic mutation data used for HotNet2 prediction (17,209 mutations from 3110 samples). Since HotNet2 did not provide prediction scores, we simply compared the number of retrieved gold-standard cancer genes for the 144 candidates predicted by HotNet2 and for the top 144 candidates predicted by MUFFINN. For MUFFINN, we performed predictions using different combinations between two networks (HumanNet and STRING) and two direct-neighbor algorithms (DNmax and DNsum). Although MUFFINN is much simpler and faster than HotNet2, we observed comparable retrieval rates for all five gold-standard cancer gene sets for the two network-based methods. Indeed, the highest performance was generally achieved by MUFFINN with DNsum, which was followed by HotNet2 and MUFFINN with DNmax (Additional file 1: Figure S8). These results also further confirm that pathway-centric approaches are superior to gene-centric methods in cancer gene prediction based on somatic mutation data. Notably, the two pathway-centric approaches, MUFFINN and HotNet2, show minimal overlap in their predictions, although they show a similar number of validated cancer genes in the gold-standard data (Additional file 1: Figure S9). These results suggest complementarity between the two pathway-centric cancer gene prediction methods and that it might be worth using both methods to maximize the discovery rate.

## Discussion

During the past few years, algorithmic research to improve cancer driver gene discovery has mostly focused on improving prediction specificity by using background mutation frequency-based models to discard false-positive predictions. However, the long-tail of the mutation frequency distribution means that frequency-based methods suffer from low sensitivity—many true positive drivers are likely discarded because their low mutation frequency cannot be distinguished from the background expectation. MUFFINN aims to improve prediction sensitivity by retrieving cancer genes with low mutation frequencies via their network associations with other cancer genes with high mutation frequencies. Although the ROC analysis results indicate higher sensitivity and specificity over state-of-the-art frequency-based methods, such as MutSigCV, MUFFINN still retrieves some likely false positive cancer genes such as TTN, which, because of their large size, accumulate many mutations. A future challenge will, therefore, be to combine the complementary features of background frequency-based and network/function-based methods to further improve the sensitivity and specificity of cancer driver gene prediction.

Correcting for gene-specific background mutation frequency has proven useful in eliminating spurious candidate driver genes during gene-centric analysis of mutation frequency data [9]. In our network-centric analysis, however, such normalization of mutation frequency did not significantly improve predictions for known cancer genes, particularly when using the mutational occurrence of all neighbors (DNsum). One possible explanation is that mutation frequency is more heterogeneous among cancer genes than amongst cancer pathways. In fact, high mutual exclusivity of mutated genes of a gene set across patients has been utilized to identify novel cancer pathways [65].

Frequently mutated cancer genes can be detected by sequencing only dozens of cancer samples. In contrast, detection of rare driver mutations may require thousands of patients. Hence, the cost-effectiveness of cancer genome projects generally rapidly decreases as the number of sequenced samples grows. A promising feature of MUFFINN is high predictive power for cancer genes using only dozens of patient samples. For the practical application of MUFFINN for sequencing-based cancer gene discovery, we implemented a user-friendly web interface (http://www.inetbio.org/muffinn/) to conduct pathway-centric analyses of mutation data by simply submitting mutation frequencies for individual genes.

Despite the successful predictions of MUFFINN, there may be room for improvements. The current algorithm uses only nonsynonymous mutations and short indels identified from whole exome sequencing. Since we anticipate an explosion of mutation data for non-coding regions, we may need to incorporate other types of mutations for both coding and non-coding regions into future developments of MUFFINN. For example, it has recently been reported that synonymous mutations [66] contribute to cancer risk and future algorithms need to account for such less well-characterized cancer-related mutations. Prediction power would, of course, also be enhanced by improving the functional networks.

## Conclusions

Here, we present a novel method for cancer gene discovery, MUFFINN, which takes into account somatic mutations in both genes and their neighbors in functional networks. We demonstrate that this pathway-centric strategy of prioritization complements conventional gene-centric mutational data analysis. Algorithm development for mutation-based cancer gene prediction has successfully dealt with false positive candidates with high background mutation frequency [24]. However, cancer gene discovery based solely on exome mutation frequency is intrinsically limited to genes with frequent mutations in coding regions. Interestingly, many known cancer genes predicted by MUFFINN with no significant evidence of somatic exonic mutations among TCGA samples are supported by their involvement in other types of genetic alterations, such as germline genetic variation, chromosomal rearrangement, and altered gene expression regulation. Ongoing efforts for the expansion of cancer genomics to whole genome sequencing and continued transcriptome and epigenome profiling will help to test whether some of these genes are targeted by regulatory rather than coding genetic variation. As demonstrated in this study, however, pathway-centric analysis of exome sequencing data and experimental follow-up may effectively fill the gap between the current status of existing data resources and the ultimate goal of completely cataloguing cancer genes in particular cancers and also in particular individuals.

## Methods

### Cancer somatic mutation data and prediction score by frequency-based methods

We used 18 types of cancer from TCGA: urothelial bladder cancer (BLCA), breast cancer (BRCA), colon and rectal adenocarcinoma (COADREAD), glioblastoma multiforme (GBM), head and neck squamous cell carcinoma (HNSC), chromophobe renal cell carcinoma (KICH), clear cell kidney carcinoma (KIRC), papillary kidney carcinoma (KIRP), acute myeloid leukemia (LAML), lower grade glioma (LGG), lung adenocarcinoma (LUAD), lung squamous cell carcinoma (LUSC), ovarian serous cystadenocarcinoma (OV), prostate adenocarcinoma (PRAD), cutaneous melanoma (SKCM), stomach adenocarcinoma (STAD), papillary thyroid carcinoma (THCA), and uterine corpus endometrial carcinoma (UCEC).

Mutational occurrence data and the prediction scores by MutSig 2.0, MutSigCV, and MutationAssessor were downloaded from GDAC (http://gdac.broadinstitute.org/runs/analyses_2014_04_16/). We downloaded TCGA data via Data Matrix (https://tcga-data.nci.nih.gov/tcga/dataAccessMatrix.htm) on May 2014, and the lists of TCGA barcodes which were used for analysis are available in the MUFFINN web application (http://www.inetbio.org/muffinn/).

### Gold-standard cancer gene sets

Because an unbiased gold-standard set of cancer genes is currently unavailable, we generated five complementary cancer gene sets derived from various sources. First, 422 cancer genes were downloaded in October 2012 from the Cancer Genome Census (CGC) database, which includes the genes for which mutations have been causally implicated in cancer [29]. While CGC is widely used as a gold-standard cancer gene set, it is heavily biased towards cancer genes derived from chromosomal translocation (>70 % of CGC genes). Since we benchmark cancer gene classifiers based on information of sequence alterations rather than structural rearrangement, we generated a second gold-standard comprising 118 cancer genes altered by point mutations, CGCpointMut. Other classes of mutations, such as translocation, large amplifications, and deletions, were excluded from CGCpointMut. The third gold-standard set was composed of 124 cancer genes based on the patterns of mutations that oncogenes are recurrently mutated at the positions while tumor suppressor genes are mutated through protein truncating alterations [1]. In particular, >20 % of the mutations in the gene need to be at recurrent positions to be classified as oncogenes and >20 % of the mutations need to be inactivated to be classified as tumor suppressor genes (20/20). The forth gold-standard set was 288 high-confidence driver genes implicated by a rule-based method (HCD) [30]. Briefly, HCD includes genes with signals of positive selection in at least two methods out of four: MuSiC [11], OncodriveFM [67], OncodriveCLUST [68], and ActiveDriver [69]. Genes which present signals of positive selection in only one method can also be included as long as additional supportive evidence is available. The fifth gold-standard set contains 797 genes identified by insertional mutagenesis in mice (MouseMut) [31, 32]. To identify new drivers of pancreatic and intestinal cancer, a mutagenic screen using Sleeping Beauty (SB) was performed and the resulting candidate cancer genes were mapped to human orthologs.

Note that we only consider 18,499 protein coding genes as MUFFINN utilizes networks such as HumanNet and STRING v10 that use protein-coding genes.

### Scoring scheme of MUFFINN

We formulated two different ways to use mutation information of direct neighbors in the network: using mutational information of only one direct neighbor with the largest number of mutations or using those of all direct neighbors. Let *M*(*i*) be the number of non-synonymous mutations of a gene (node) i across the set of individuals and *W*(*i, j*) be the normalized edge weights between gene *i* and gene *j*. Then, the raw scores of gene *i* by MUFFINN are defined as follows.

DNmax: max of the direct neighbor mutational occurrences:

$$ {f}_{DNmax}\left(\mathrm{i}\right)=M\left(\mathrm{i}\right)+\underset{j}{ \max }M\left(\mathrm{j}\right)*W\left(\mathrm{i},\mathrm{j}\right) $$

DNsum: sum of the direct neighbor mutational occurrences:

$$ {f}_{DNsum}\left(\mathrm{i}\right)=M\left(\mathrm{i}\right)+{\displaystyle \sum_j}\left\{\frac{\mathrm{M}\left(\mathrm{j}\right)}{\mathrm{Deg}\left(\mathrm{j}\right)}\right\},\kern1em j: neighbors\  of\  node\;i $$

where Deg(*j*) is the number of network neighbors of gene i. We found that accounting for edge weights increases the prediction performance when using DNmax but decreases the performance when using DNsum. The calculated scores using the above equations are then transformed into probability scores based on logistic regression.

For taking into account mutations in indirect network neighbors, we used three distinct network diffusion algorithms, Gaussian smoothing (GS), random walk with restart (RWR), and iterative ranking (IR).

In the GS algorithm, labels are propagated by Gaussian probability density functions with the aim of finding optimal solutions to minimize two differences: (i) between the initial and final scores of a labeled node; (ii) between the label score of a node and each of its neighbors [33].

$$ f={\mathrm{argmin}}_f\kern0.5em \alpha \cdot {\displaystyle {\sum}_i{\left(\mathrm{f}\left(\mathrm{i}\right)-{\mathrm{f}}^0\left(\mathrm{i}\right)\right)}^2+\left(1-\upalpha \right)\cdot {\displaystyle {\sum}_i{\displaystyle {\sum}_j{W}_{ij}\left(\mathrm{f}\left(\mathrm{i}\right)-\mathrm{f}\left(\mathrm{j}\right)\right),}}\ }\kern0.2em \left(\mathrm{node}\ \mathrm{j}\ \mathrm{are}\ \mathrm{neighbors}\ \mathrm{of}\ \mathrm{node}\ \mathrm{i}\right) $$

While a binary score, 0 or 1, is generally used as the initial score in GS, we modified *f*^0^ to take into account the mutation occurrence score. We ran network diffusion based on the Gaussian smoothing algorithm using geneMANIA [70] software.

In the RWR algorithm, the (1 − *α*) portion of the node scores at time *t* are iteratively propagated to neighbors based on the adjacency matrix *U* of which columns are normalized [26].

$$ {f}^{t+1}=\upalpha \cdot {f}^0+\left(1-\upalpha \right)\cdot U{f}^t,\kern1em \left(U\  is\  column\  normalized\  adjacency\  matrix\right) $$

The IR algorithm is similar to RWR while a conditional probability matrix among nodes is used instead of column-normalized matrix *U*.

$$ {f}^{t+1}=\alpha \cdot {f}^0+\left(1-\upalpha \right)\cdot {\displaystyle {\sum}_jp\left(\mathrm{i}\Big|\mathrm{j}\right){\mathrm{f}}^{\mathrm{t}},\kern1em \Big(\left(p\left(i\Big|j\right)\  is\  the\  conditional\  probability\  of\  arriving\  nodes\ i\  from\ j.\right)} $$

For RWR and IR, we used NetWalk and NetRank, respectively, available in the GUILD software [71]. All software for network diffusion was run with default parameter settings.

For MUFFINN analysis with normalized mutation frequency by BMR, the negative logarithm with base 10 of MutsigCV scores was used as the initial node scores instead of the mutational occurrences.

### Selection of MUFFINN-specific candidate genes for validation

Several criteria were applied for candidate gene selection. First, the number of neighbors with mutations should be more than one. This criterion can avoid false positives caused by a few hub genes which have high mutation occurrences, affecting many connected neighbors. Second, the genes should be ranked within the top 1000 by MUFFINN (either by DNmax or DNsum of the neighbor mutational occurrences) with high probability (>0.5), yet ranked below 1000 with poor *P*-values (>0.5) for gene-centric methods (Mutsig2.0, MutsigCV, or MutationAssessor). At the same time, we focused on the genes whose ranks differed greatly (>1000) between MUFFINN and gene-centric methods. Lastly, we excluded known cancer genes based on 422 cancer genes by CGC [29] and 124 cancer genes by the mutational patterns of the 20/20 rule [1], the two most well-known and most confident cancer gene sets. This filtration ensured that the only novel candidates were included in our candidate gene set for validation using literature review.

## Additional file

Additional file 1: Supplementary online information including supplementary Figures S1–S9 and supplementary Table S1. (PDF 4546 kb)
