# Supplementary material for: MUFFINN: cancer gene discovery via network analysis of somatic mutation data
Source: Genome Biol. 2016 Jun 23;17:129. doi: 10.1186/s13059-016-0989-x (PMC4918128; doi:10.1186/s13059-016-0989-x)
Supplement: Additional file 1: — Supplementary online information including supplementary Figures S1–S9 and supplementary Table S1. (PDF 4546 kb) [file 13059_2016_989_MOESM1_ESM.pdf]

Supplementary Online Information

MUFFINN: cancer gene discovery via network analysis of somatic mutation data

Ara Cho, Jung Eun Shim, Eiru Kim, Fran Supek, Ben Lehner and Insuk Lee

Contents

Supplementary Fig. S1

Supplementary Fig. S2

Supplementary Fig. S3

Supplementary Fig. S4

Supplementary Fig. S5

Supplementary Fig. S6

Supplementary Fig. S7

Supplementary Fig. S8

Supplementary Fig. S9

Supplementary Table S1

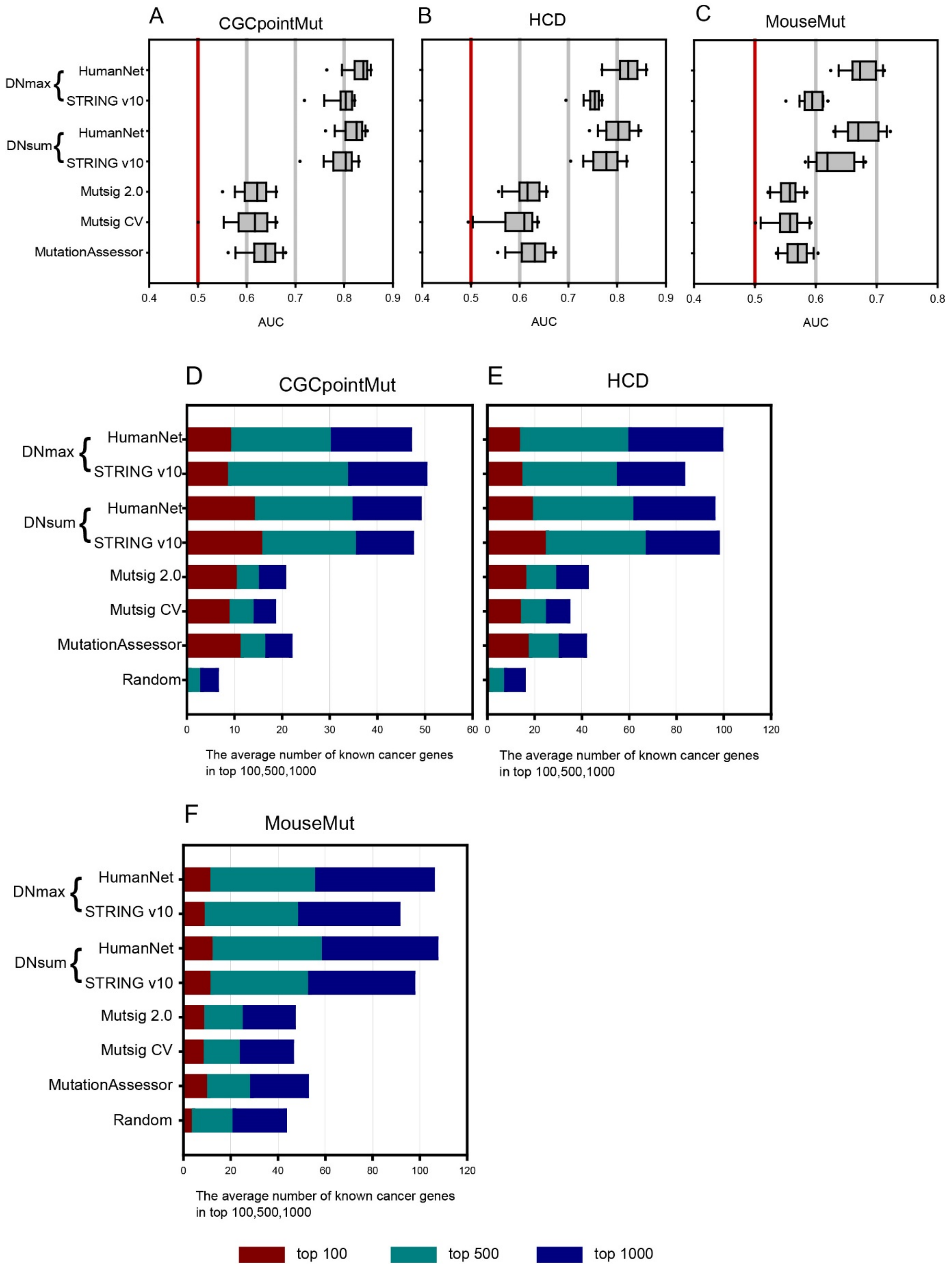

Supplementary Figure S1. Distribution of AUC for 18 types by MUFFINN using direct neighbour (DNmax and DNsum), Mutsig 2.0, Mutsig CV, and MutationAssessor based on cancer genes annotated by (A) CGCpointMut, (B) HCD, and (C) MouseMut. The average number of retrieved known cancer genes in top 100, 500, 1000 candidates among 18 types by MUFFINN using direct neighbours, Mutsig, and MutationAssessor based on cancer genes annotated by (D) CGCpointMut, (E) HCD, and (F) MouseMut.

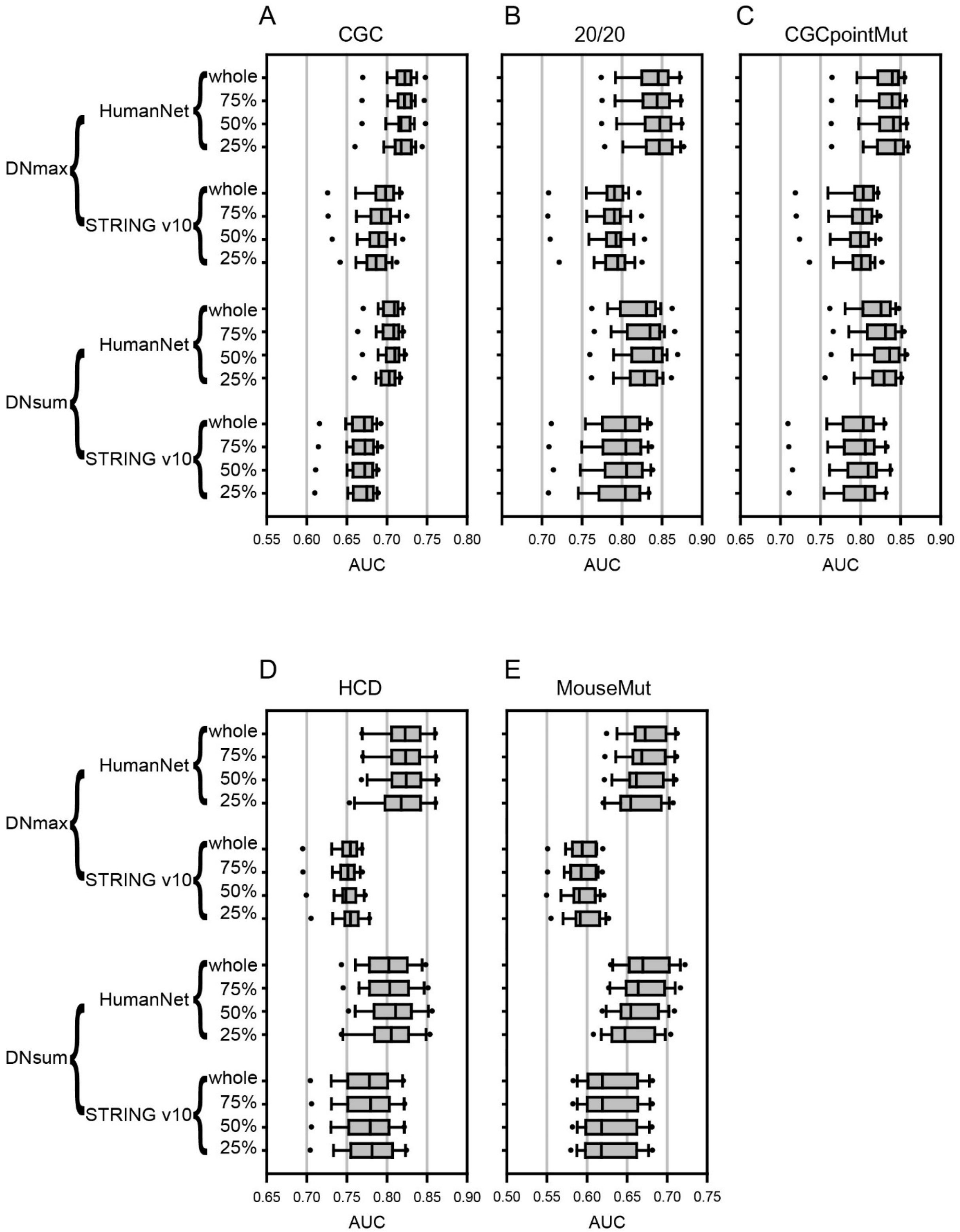

Supplementary Figure S2. Distribution of AUC for 18 types by MUFFINN using direct neighbour (DNmax and DNsum) with top 75%, 50%, 25%, or whole of network links, based on cancer genes annotated by (A) CGC, (B)20/20 rule, (C) CGCpointMut, (D) HCD, and (E) MouseMut.

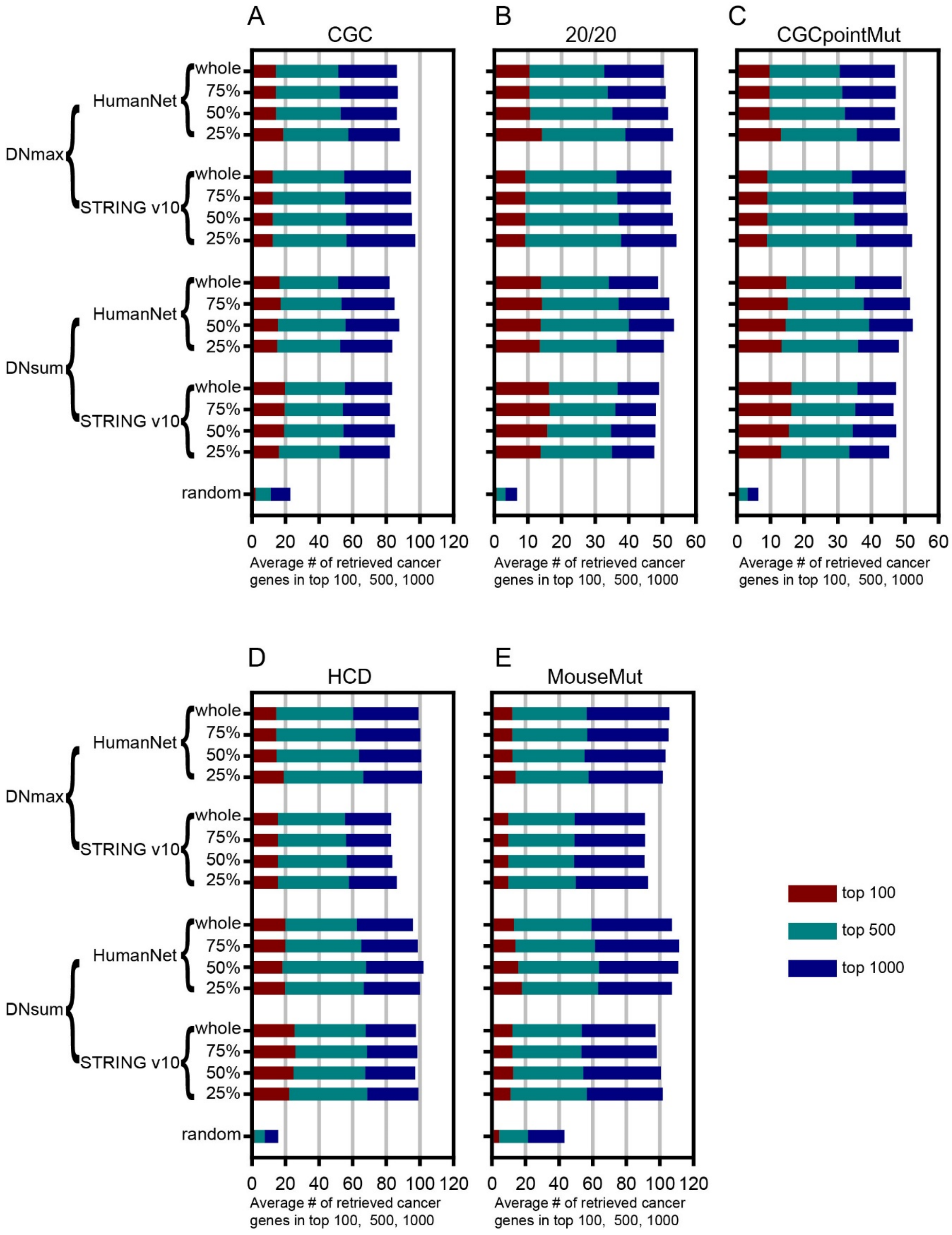

Supplementary Figure S3. The average number of retrieved known cancer genes in top 100, 500, 1000 candidates among 18 types by MUFFINN using direct neighbour (DNmax and DNsum) with top 75%, 50%, 25%, or whole of network links, based on cancer genes annotated by (A) CGC, (B)20/20 rule, (C) CGCpointMut, (D) HCD, and (E) MouseMut.

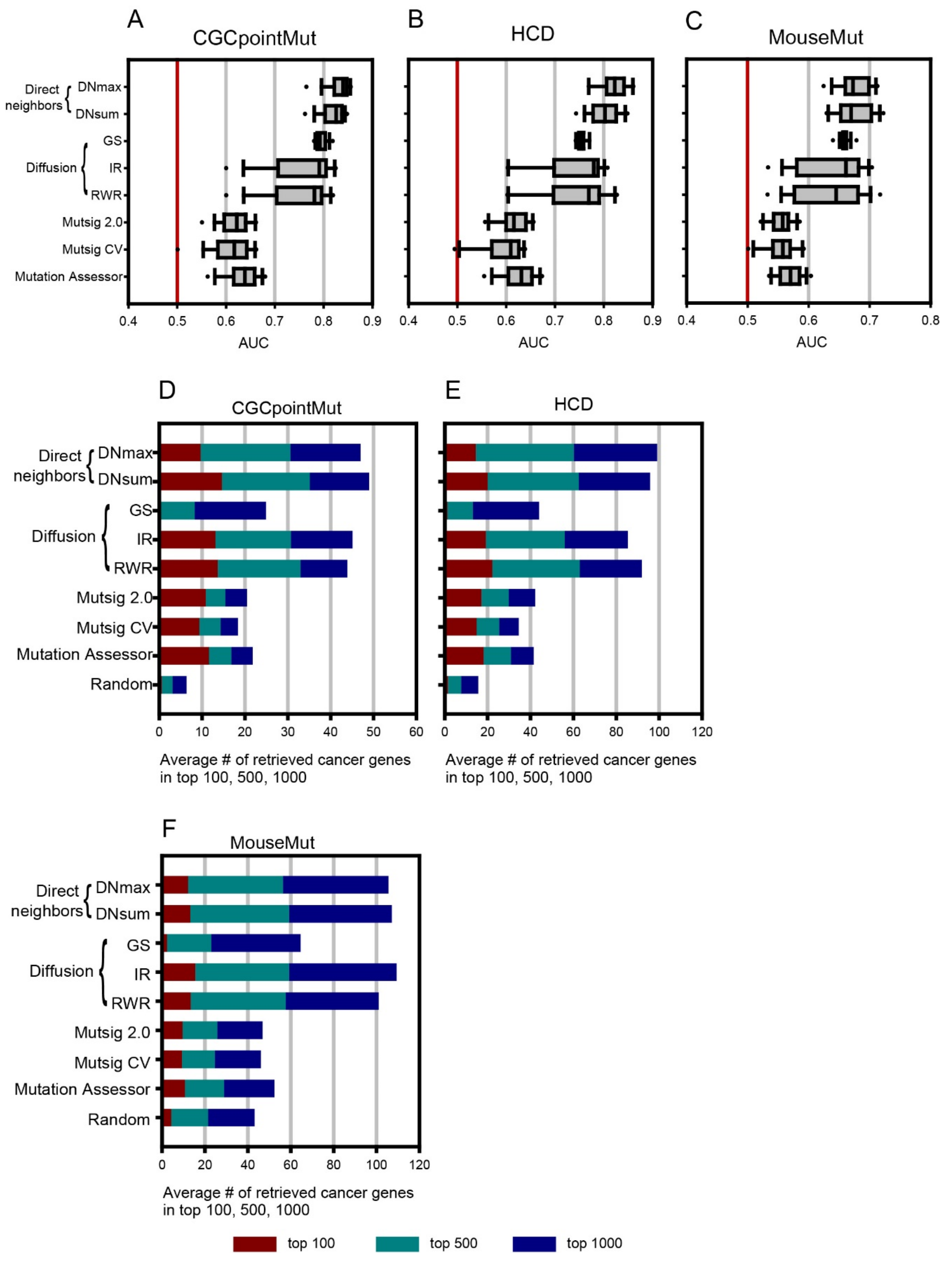

Supplementary Figure S4. Distribution of AUC for 18 types by MUFFINN with five different network algorithms, two of them consider direct neighbours only and three of them consider all genes of the network by network diffusion, on HumanNet based on cancer genes annotated by (A) CGCpointMut, (B) HCD, and (C) MouseMut. The average number of retrieved known cancer genes in top 100, 500, 1000 candidates among 18 types by MUFFINN with the same five network algorithms on HumanNet based on cancer genes annotated by (D) CGCpointMut, (E) HCD, and (F) MouseMut.

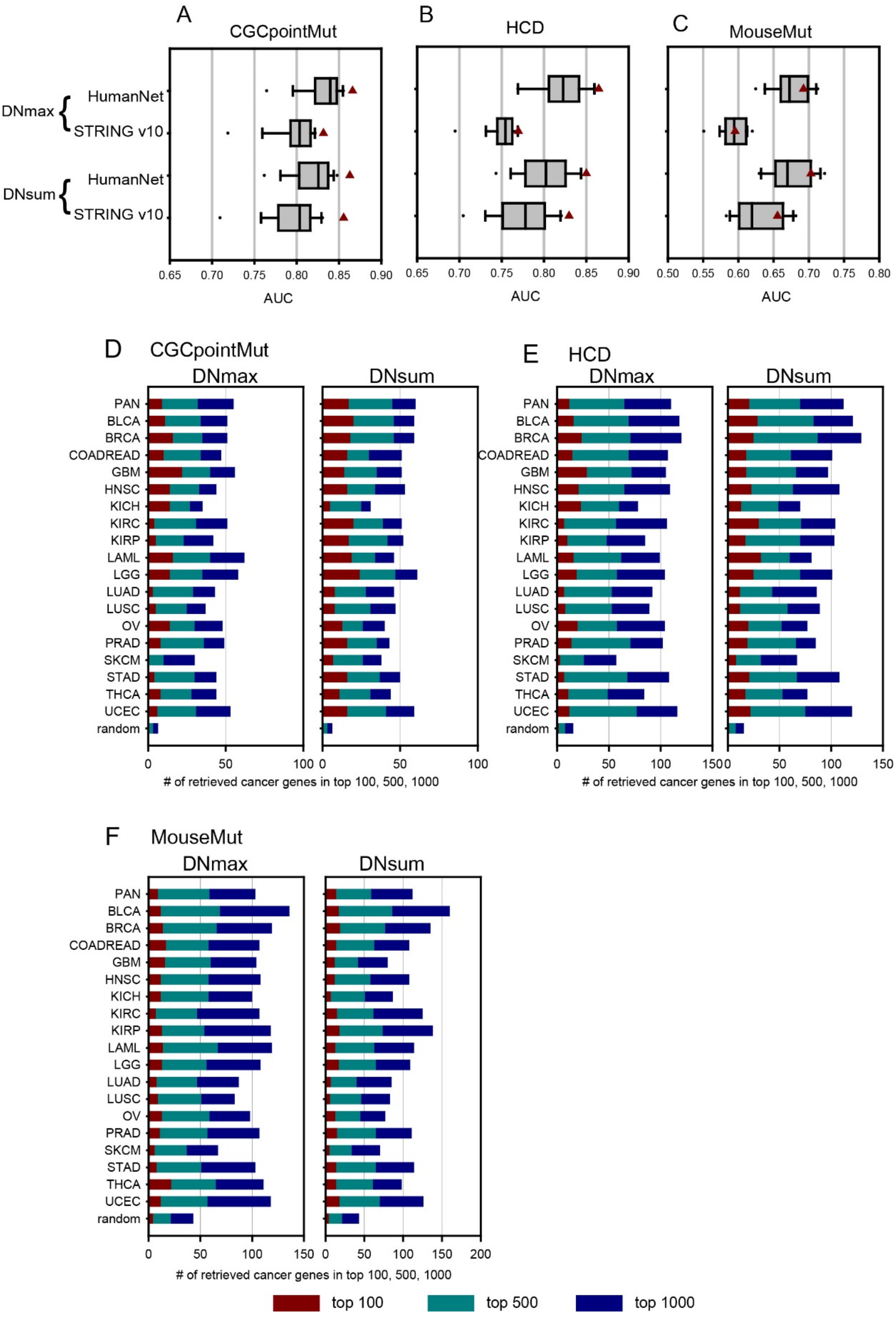

Supplementary Figure S5. Distribution of AUC for 18 type and the pan-cancer sample (red triangular dots) by MUFFINN using direct neighbours on HumanNet based on cancer genes annotated by (A) CGCpointMut, (B) HCD, and (C) MouseMut. The number of retrieved known cancer genes in top 100, 500, 1000 candidates for the pan-cancer and 18 type samples by MUFFINN using direct neighbours on HumanNet based on cancer genes annotated by (D) CGCpointMut, (E) HCD, and (F) MouseMut.

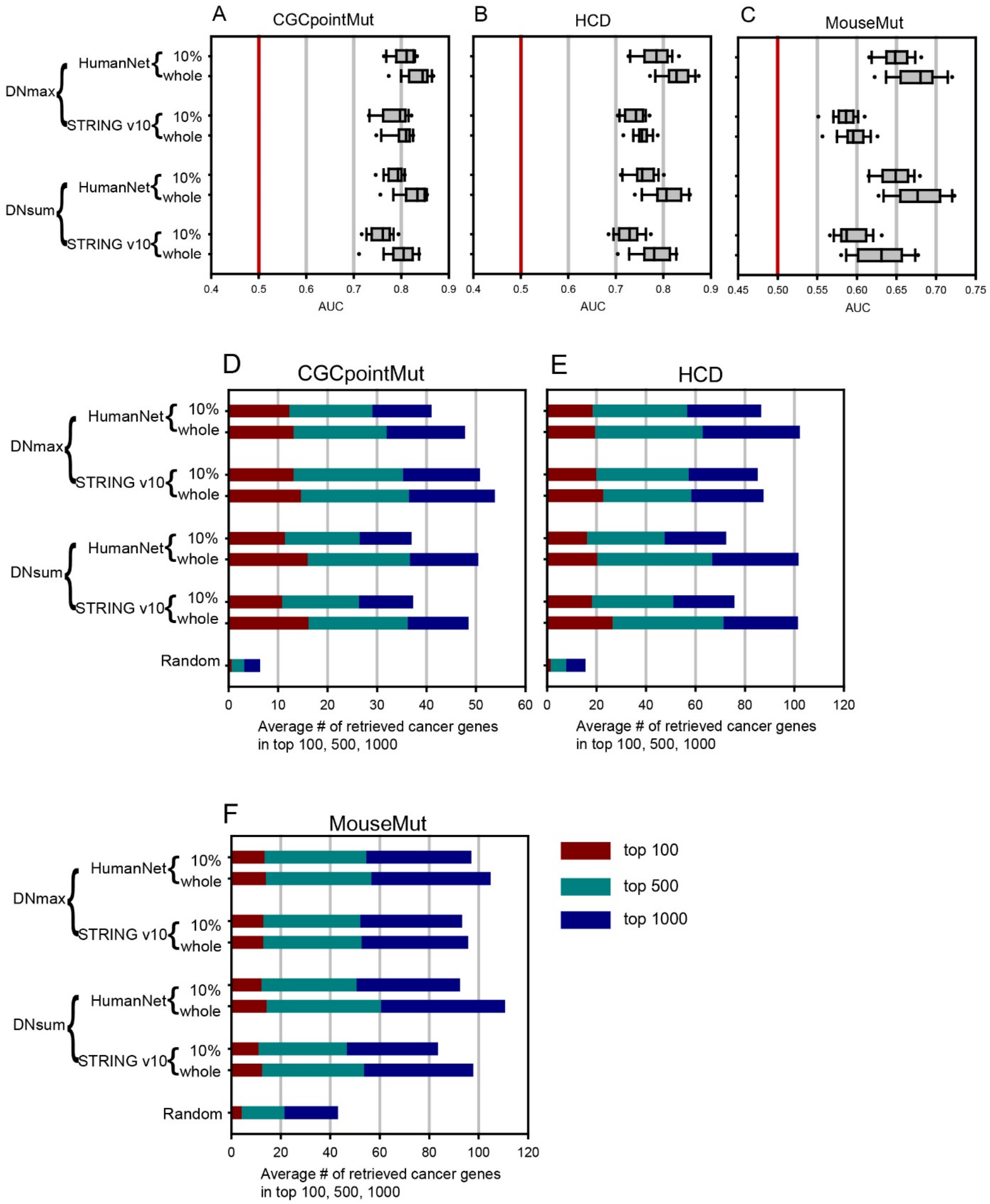

Supplementary Figure S6. Comparison of AUC distribution for 18 types by MUFFINN using direct neighbours between using whole patient and 10% samples based on cancer genes by (A) CGCpointMut, (B) HCD, and (C) MouseMut. Each box plot for 10% sample is based on 18 average AUC scores of 100 random sampling for each of 18 types, and that for whole sample is based on 18 AUC scores for each type. Comparison of the number of retrieved known cancer genes in top 100, 500, 1000 candidates for 18 types by MUFFINN using direct neighbours between using whole patient and 10% samples based on cancer genes by (D) CGCpointMut, (E) HCD, and (F) MouseMut. Each box plot for 10% sample is based on 18 average AUC scores of 100 random samples and that for whole sample is based on 18 AUC scores for each type.

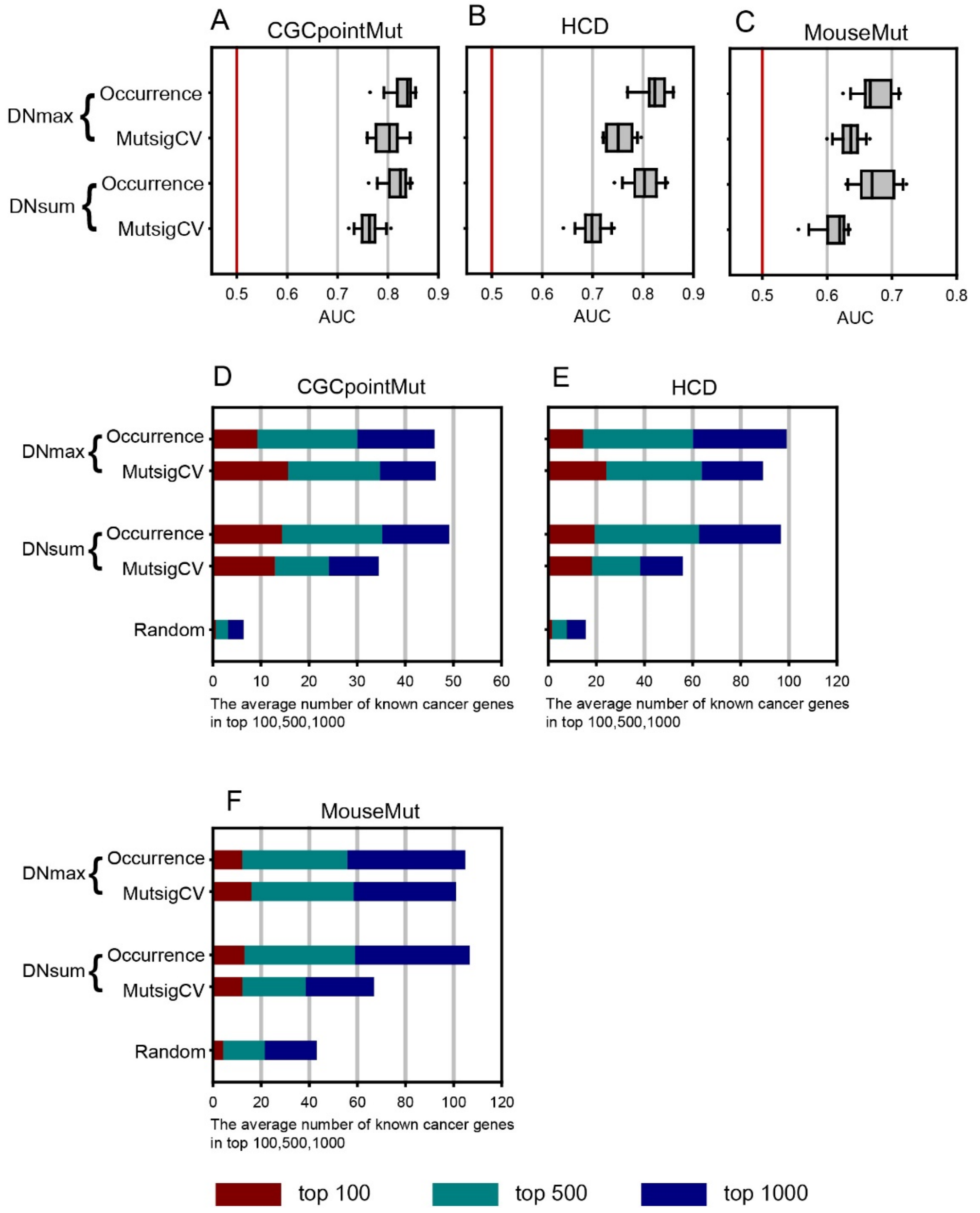

Supplementary Figure S7. MUFFINN was performed with raw mutation frequencies or MutsigCV scores, which are normalized by BMR and mutational heterogeneity. HumanNet was used as network model. Comparison of predictions for cancer genes annotated by (A) CGCpointMut, (B) HCD, and (C) MouseMut. AUC scores for 17 cancer types (LAML was excluded in the analysis due to the indiscriminative MutsigCV scores by low mutation rate in leukemia) are represented as box plot. Performance comparisons were also conducted based on the number of retrieved cancer genes annotated by (D) CGCpointMut, (E) HCD, and (F) MouseMut in top 100, 500, 1000 candidates.

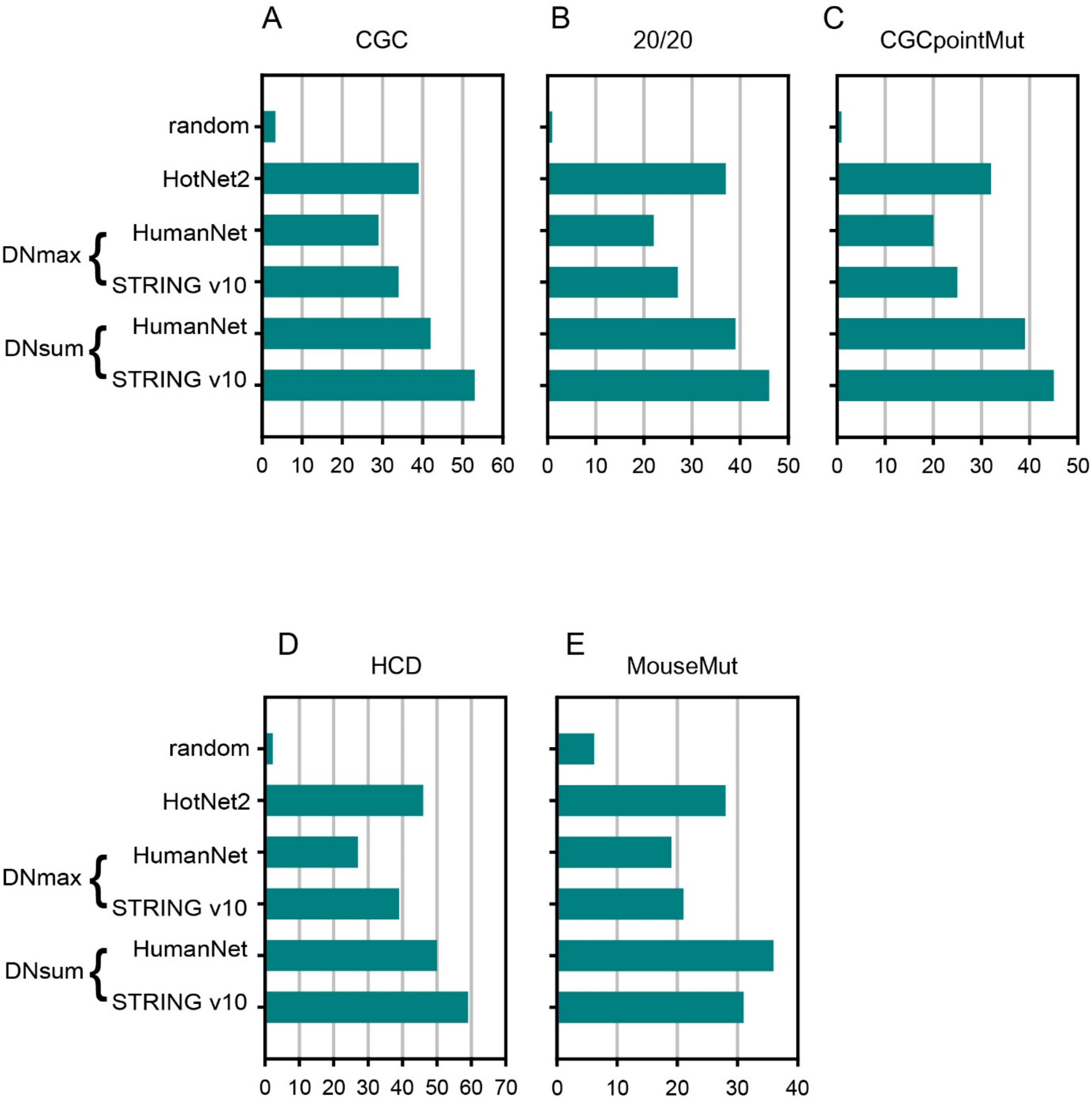

Supplementary Figure S8. Comparison between HotNet2 and MUFFINN predictions. The number of retrieved known cancer genes in 144 candidates by HotNet2 and top 144 candidates by MUFFINN using direct neighbour (DNmax and DNsum) with two different networks, based on cancer genes annotated by (A) CGC, (B)20/20 rule, (C) CGCpointMut, (D) HCD, and (E) MouseMut.

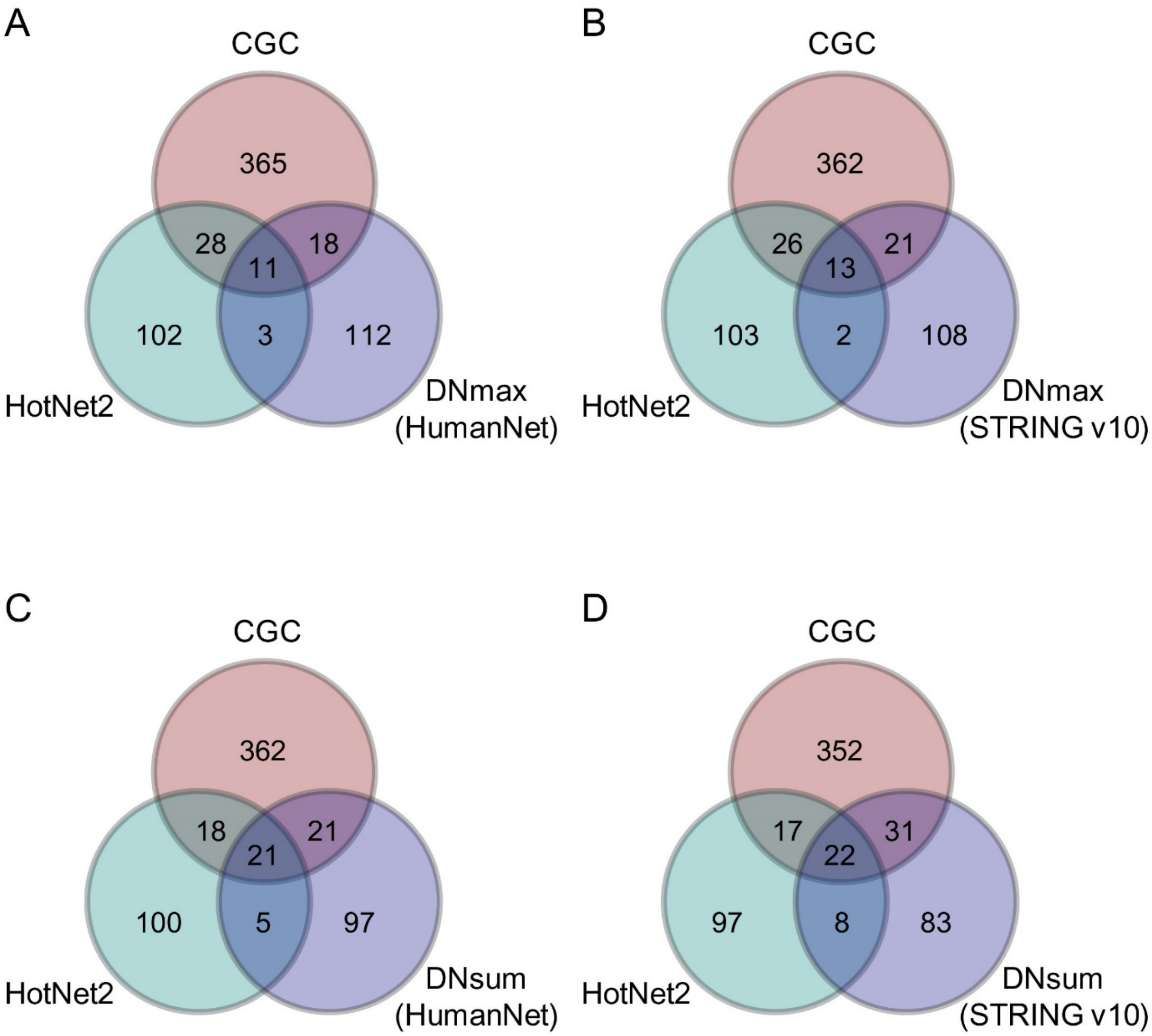

Supplementary Figure S9. Venn diagram among 422 gold-standard cancer genes by CGC, 144 cancer gene candidates by HotNet2, and top 144 candidates by MUFFINN using (A) DNmax and HumanNet, (B) DNmax and STRING v10, (C) DNsum and HumanNet, (D) DNsum and STRING v10.

Supplementary Table S1. 199 candidate cancer genes not identified by gene-centric methods but prioritised by MUFFINN.

Gene symbol	Supportive cancer type (Key type is marked by underline)	DNmax score (for the key type)	DNsum score (for the key type)	Class	Evidence/Implication	Ref.
TTN	COADREAD,KIRC,LGG,THCA,UCEC	0.9995	1.0000	5		
NEB	BLCA,COADREAD,GBM,KIRP,PRAD,UCEC	0.9991	0.8105	4	Nebulin(protein) was also strongly expressed in the pancreatic juice of pancreatic cancer patients.	[1]
MAEA	KICH	0.9738	0.3842	5		
ISM2	KICH	0.9710	0.3679	5		
CACNA1A	BLCA,COADREAD,GBM,HNSC,LUAD,SKCM,STAD	0.9707	0.3339	4	It was identified as novel tumor suppressor candidates methylated in lung tumors.	[2]
SNED1	KICH,KIRP,STAD	0.9596	0.4316	4	High expression of SNED1 correlates with poor outcome for ER(-)/PR(-) breast cancer patient.	[3]
OBSCN	BLCA,KIRP,LUSC,PRAD,STAD,UCEC	0.9495	0.8506	2	A germline nucleotide variant of OBSCN could be involved in cancer predisposition.	[4]
MYOM2	BRCA,COADREAD,KIRP,LUSC,PRAD,STAD,UCEC	0.9431	0.1309	4	Downregulated in breast cancer	[5]
ANK1	COADREAD,HNSC,LUSC,STAD,UCEC	0.9370	0.1858	3	Mapped to deleted region in colorectal cancer	[6]
ANKRD1	BRCA,HNSC,KIRP,LUSC,PRAD,STAD	0.9199	0.0817	4	High expression of ANKRD1 in ovarian carcinoma is associated with poor survival.	[7]
MYBPC3	BLCA,BRCA,COADREAD,HNSC,STAD	0.9104	0.0822	5		
ANKRD23	BLCA,COADREAD,HNSC,LUAD,LUSC	0.8990	0.0603	5		
IGF1R	BRCA,UCEC	0.8984	0.2164	4	miR-133a functions as a tumor suppressor in gastric cancer by repressing IGF1R	[8]
VBP1	KIRC	0.8954	0.0774	4	Differentially expressed in the uveal melanoma cell lines	[9]

DNMT3L	LAML	0.8954	0.1886	4	DNA methylation profile at DNMT3L promoter is a promising biomarker for cervical cancer.	[10]
MAPK1	THCA	0.8924	0.2236	4	Tumor-suppressive role of miR-378 might be achieved by the direct interaction with MAPK1.	[11]
MAP2K2	THCA	0.8872	0.1207	1	Newly added to CGC	[12]
RBX1	KIRC	0.8840	0.0779	4	Aberrant expression of RBX1 is correlated to gastric cancer patient survival time.	[13]
COQ7	BRCA	0.8610	0.0648	5		
MYOM1	BLCA,COADREAD,KIR`P,LUAD,LUSC,PRAD,STAD	0.8373	0.1385	3	One of the targets of the viral DNA integration for the hepatitis B virus (HBV)-related hepatocellular carcinomas (HCCs).	[14]
PRG4	COADREAD,KIRP,LUAD,LUSC,STAD,UCEC	0.8258	0.1136	5		
HIST3H3	LAML	0.8188	0.1007	5		
PPP2R2A	THCA	0.8178	0.1272	3	Somatic deletion of PPP2R2A plays an important role in prostate cancer susceptibility.	[15]
REST	BLCA,COADREAD,KIRP,PRAD,STAD,UCEC	0.8143	0.1072	1	REST acts as a tumor suppressor and plays an oncogenic role based on the type of cancer.	[16]
NCL	LAML	0.8129	0.1052	4	NCL regulates the expression of a specific subset of miRNAs which are causally involved in breast cancer initiation, progression, and drug resistance.	[17]
MYH6	BLCA,COADREAD,LUSC,UCEC	0.8090	0.1457	5		
PPM1K	COADREAD,KIRP,LAML,THCA,UCEC	0.8077	0.2561	5		
USP33	KIRC	0.8068	0.1538	4	USP33 suppresses tumor by mediating the inhibitory function of	[18]

					Slit-Robo signaling on for colorectal cancer cell migration.	
CSF2RB	LAML	0.8038	0.1515	2	CSF2RB rs16997517 was directly related to pollen allergies in controls and to reduced squamous cell cervical cancer risk.	[19]
AEBP1	BLCA,LUSC,PRAD,STAD,UCEC	0.7942	0.1861	4	Its promoters are densely methylated in the rat prostate cancer cell lines.	[20]
YWHAB	THCA	0.7938	0.1668	1	It is known to play a key role in cellular proliferation and oncogenic transformation and has been reported to be involved in the development of breast cancer.	[21]
MCC	COADREAD	0.7818	0.1433	1	MCC mutations can drive colon carcinogenesis in the mouse and in vitro experiments.	[22]
CYCS	KICH	0.7790	0.3876	5		
MAP1B	BLCA,KIRP,LUSC,PRAD	0.7782	0.1990	5		
FLT4	LAML	0.7754	0.1433	1	Newly added to CGC	[12]
MYH7	BLCA,COADREAD,LUSC,STAD,UCEC	0.7709	0.2417	5		
CCT3	KIRC	0.7558	0.0743	4	Its expression is significantly elevated in hepatocellular carcinoma.	[23]
CAB39	KIRC	0.7549	0.0691	4	Known to play a critical role in regulating a biochemical balance between rapid proliferation and invasion in the presence of metabolic stress in microenvironment in glioblastoma.	[24]
UBE2I	GBM,HNSC,KICH,LAML,LGG,OV	0.7544	0.0888	4	Showed distinct expression patterns in histological types of epithelial ovarian cancer.	[25]

TCAP	COADREAD,LUAD,LUSC,STAD,UCEC	0.7346	0.0520	3	Belongs to amplicon on chromosome 17q21 which is frequently amplified in breast tumor.	[26]
PPM1B	COADREAD,KIRP,LAML,PRAD,UCEC	0.7313	0.2854	5		
ARHGEF7	LAML	0.7296	0.1368	4	Arhgef7 protein is a positively regulate Hippo pathway of which inactivation contribute to cancer.	[27]
ZBTB18	LAML	0.7269	0.1891	5		
CAPN3	COADREAD,KIRP,STAD,UCEC	0.7111	0.1160	4	Activated Capn3 leads to the overexpression of E2F3, which in turn could be responsible for urothelial tumor cell proliferation.	[28]
LIF	LAML	0.7102	0.1308	1	It is known to play an important role in inducing cancer cachexia.	[29]
HIF1A	BLCA,BRCA,GBM,HNSC,LGG,OV	0.7102	0.0754	2	Polymorphism in HIF1A contribute to the risk of digestive tract cancer	[30]
GRB2	GBM,KIRP,LAML,THCA	0.7091	0.1829	4	Key molecule in intracellular signal transduction and a therapeutic target for solid tumor invasions and metastasis	[31]
UBE3A	BRCA,HNSC,LGG,OV	0.7049	0.0829	4	UBE3A plays an important role in negating the consequences of p53 activation on ERK pathway which are often deregulated in cancer	[32]
IRS2	BRCA	0.7012	0.0815	4	Contribute to tumor progression in Pten(+/-) mice by stimulating both Myc and DNA synthesis	[33]
IL3	LAML	0.6827	0.1426	3	IL3/IgH fusion is identified in cutaneous lymphoma cell	[34]
MDH1	LGG	0.6810	0.1011	5		
PPM1L	BLCA,KIRP,LAML,PRAD,STAD,THCA	0.6802	0.1949	4	The 3'UTR of PPM1L transcript was down-	[35]

					regulated more than two-folds in tumors compared to matched mucosa.	
LIN7C	GBM	0.6799	0.0680	5		
NR1I2	BRCA	0.6797	0.0885	2	Common polymorphisms of regulator gene NR1I2 could be implicated in lymphoma risk.	[36]
SPTBN4	BLCA,COADREAD,KIRP,UCEC	0.6786	0.2689	5		
TRDMT1	LAML	0.6773	0.2000	4	Aberrant tRNA post-translational modification including methylation has been implicated in cancer, and TRDMT1 methylates tRNA.	[37]
WISP2	LAML	0.6687	0.1895	1	WISP-2 is relevant to tumorigenesis and malignant transformation, particularly in breast cancer, colorectal cancer and hepatocarcinoma.	[38]
CASK	GBM	0.6630	0.1061	4	CASK overexpression is associated with an unfavorable prognosis in colorectal cancer.	[39]
APBA2	GBM	0.6630	0.0781	4	Aberrant APBA2 methylation predict peritoneal micrometastasis for gastric cancer patients.	[40]
MYH3	BLCA,STAD,UCEC	0.6582	0.2644	5		
IDH3G	LGG	0.6521	0.0951	5		
IDH3A	LGG	0.6382	0.0971	4	Differentially expressed (down regulated) by chromosomal aneuploidy affect in colorectal cancer cells.	[41]
USP7	BRCA,PRAD	0.6343	0.0979	4	Plays an important role in the regulation of lung cancer development	[42]
ACTN1	BLCA,COADREAD,KIRP,STAD,UCEC	0.6291	0.1478	4	Is known as tumor specific splice variant in many type of cancer	[43]

CALM1	BRCA,COADREAD,KIRP,UCEC	0.6277	0.6050	4	Differentially Expressed in non-small cell lung cancer	[44]
MYH4	BLCA,COADREAD,STAD,UCEC	0.6269	0.2019	5		
HDAC1	LAML	0.6224	0.3091	1	Regulates cell proliferation in human myeloid leukemia cell	[45]
MYH8	BLCA,COADREAD,STAD,UCEC	0.6159	0.1917	5		
FHL2	BLCA,COADREAD,STAD,UCEC	0.6126	0.1222	4	Interacts with a variety of transcription factors known to be involved in tumor development and expression in deregulated in cancer.	[46]
PTPN6	LAML	0.6069	0.1488	4	Both the PTPN6 transcripts and proteins were overexpressed in ovarian epithelial carcinoma cell lines	[47]
APEX1	BLCA,GBM,HNSC	0.6058	0.0853	2	Germline variants in APEX1 increases the risk of cancer development by contributing apurinic/apyrimidinic site accumulation in DNA.	[48]
NRF1	BLCA,STAD,UCEC	0.6052	0.0737	4	Regulate NOR1 promoter which is a candidate tumor suppressor gene.	[49]
ATR	BLCA,BRCA,GBM,OV	0.5998	0.2001	1	Newly added to CGC	[12]
TP53BP2	HNSC,OV	0.5977	0.0757	5		
GALNT11	KICH	0.5972	0.3700	5		
HOXA5	COADREAD	0.5928	0.0747	4	miR-1271 regulates non-small-cell lung cancer cell proliferation and invasion, via the down-regulation of HOXA5.	[50]
TRIM63	BLCA,COADREAD,STAD,UCEC	0.5926	0.0656	5		
ING1	BRCA,HNSC	0.5918	0.0860	4	Recently reported as a validated target of a miRNA let-7b which suppresses gastric cancer malignancy	[51]

PPM1A	STAD	0.5912	0.2530	4	Loss of PPM1A expression enhances invasion and epithelial-to-mesenchymal transition in cancer.	[52]
ELK1	THCA	0.5898	0.1847	4	Binding of Est1 to CIP2A promoter is required for expression of CIP2A which has been identified as proto-oncogene in various types of human cancer.	[53]
PIN1	BLCA,BRCA,OV	0.5868	0.0917	4	Prevalently over-expressed in kinds of breast cancer cell lines and tissues.	[54]
MYH2	BLCA,COADREAD,STAD	0.5830	0.2302	5		
CHEK1	HNSC,OV	0.5822	0.0848	4	CHEK1 protein levels were significantly associated with higher tumor stage, higher mitotic index, pleomorphism and lymphovascular invasion.	[55]
EFEMP2	BLCA,GBM,OV	0.5818	0.0733	2	SNP in the EFEMP2 gene was associated with increased risk in breast cancer.	[56]
PRMT1	BRCA,HNSC	0.5817	0.0926	4	Overexpressed in Non-Small Cell Lung Carcinomas and are involved in proliferation in lung cancer	[57]
SREK1	LAML	0.5811	0.1085	5		
XYLB	COADREAD,KIRP,STAD	0.5808	0.0903	5		
SH3BP2	LAML	0.5801	0.1309	5		
SIRT1	BLCA,BRCA,OV	0.5795	0.1070	1	SIRT1 could act as either a tumor suppressor or tumor promoter depending on its targets in specific signaling pathways or in specific cancers.	[58]
RPS6	LAML	0.5781	0.1102	4	Reduced Rps6 phosphorylation and activation of p53 results	[59]

					in selective elimination of leukemia cells.	
NOP2	LAML	0.5757	0.2351	5		
ARHGEF4	COADREAD	0.5744	0.0736	4	Inactivation of ARHGEF4 can promote tumorigenesis.	[60]
GK	BLCA,COADREAD,STAD	0.5710	0.1078	5		
GK2	STAD	0.5710	0.0849	5		
EPS8	GBM	0.5700	0.0745	4	Overexpressed in >60% of human breast cancer sample and knock down of Eps8 inhibits breast cancer cell migrations	[61]
GK5	BLCA,COADREAD,STAD	0.5682	0.0593	5		
E4F1	HNSC	0.5678	0.0739	5		
MRI1	LGG	0.5662	0.0996	5		
C1QBP	LGG	0.5657	0.1086	4	Critical for prostate cancer cell proliferation and may be a novel marker of clinical progression in prostate cancer.	[62]
CAT	BRCA	0.5639	0.0692	2	SNPs of CAT have limited associations with the risk of prostate cancer in Chinese populations.	[63]
COL4A5	GBM	0.5638	0.1568	5		
FGGY	BLCA,UCEC	0.5616	0.0735	5		
AK2	LGG	0.5614	0.1044	4	AK2 deficiency enhances cell proliferation and induces tumor formation.	[64]
CKS1B	THCA	0.5614	0.1263	4	Frequently overexpressed in different tumor subtypes.	[65]
STK4	HNSC	0.5604	0.0660	4	miR-18a acts as an oncomiR targeting STK4 in prostate cancer.	[66]

SUV39H1	LAML	0.5601	0.2080	4	Pin1-mediated reduction of SUV39H1 stability contributes to convey oncogenic signals for aggressiveness of human breast cancer.	[67]
ILKAP	LAML	0.5600	0.1858	3	Frequently observed allelic loss at chromosome 2q37 regions contains ILKAP	[68]
MYH13	BLCA,COADREAD,STAD,UCEC	0.5593	0.1567	5		
YWHAZ	KIRP,THCA,UCEC	0.5586	0.2410	4	YWHAZ knock-down significantly affected cell proliferation and migration in the prostate cancer cells.	[69]
TGFBR1	UCEC	0.5584	0.2971	3	Tgfbr1 haploinsufficiency was associated with early onset adenocarcinoma and increased tumor cell proliferation.	[70]
MYH1	BLCA,COADREAD	0.5580	0.1696	5		
DCC	HNSC	0.5575	0.2794	4	Known as putative candidate tumor suppressor and inactivated by promoter hypermethylation in cancer	[71]
TP53BP1	BLCA,GBM	0.5575	0.1184	2	TP53BP1 rs2602141 A/C was associated with cancer risk in Asians.	[72]
YWHAQ	THCA	0.5558	0.1408	5		
EMILIN1	BLCA,UCEC	0.5537	0.0923	5		
DDB2	COADREAD,STAD	0.5535	0.0751	4	Overexpressing DDB2 in human ovarian cancer cells suppressed its capability to recapitulate tumors in athymic nude mice	[73]
PLK1	LAML	0.5512	0.1433	1	known as oncoprotein in leukemia	[74]
ANKRD2	HNSC,STAD	0.5492	0.0604	5		
DMBT1	GBM	0.5491	0.1243	3	DMBT1 expression is frequently lost in lung	[75]

					cancer due to gene deletion, suggesting that inactivation of DMBT1 may play an important role in lung tumorigenesis.	
MUC4	KIRP	0.5479	0.8303	4	MUC4 is an important tumor marker overexpressed in lung cancer and uniquely expressed in pancreatic ductal adenocarcinoma.	[76]
MYBPC1	BLCA,UCEC	0.5447	0.0877	4	MYBPC1 mRNA expression were highly correlated between CUB (contralateral unaffected breasts) and matched tumor samples.	[77]
HIBADH	LGG	0.5440	0.1011	5		
PARP1	BRCA,HNSC	0.5420	0.1853	4	PARP1 expression was correlated to clinicopathological variables, outcome and expression of other key DNA repair proteins expression in breast cancer.	[78]
ERCC3	BLCA,OV	0.5298	0.0756	5		
S100B	OV	0.5294	0.0743	4	S100B promotes glioma growth.	[79]
SSTR3	OV	0.5268	0.0701	4	The expression of SSTR3 protein was significantly lower in gastric cancer compared with normal mucosa.	[80]
ACADSB	LGG	0.5266	0.1058	5		
IRS1	BRCA	0.5255	0.1031	4	miR200c plays crucial roles in prostate cancer by post-transcriptional regulation of IRS1.	[81]
SYNE1	BLCA,BRCA,COADREAD,KIRC,STAD,UCEC	0.5247	0.9554	5		
PPM1G	LAML	0.5242	0.1970	4	PPM1G downregulate USP7S which plays a major role in regulating genome stability and cancer prevention by controlling the key	[82]

					proteins involved in the DNA damage response.	
FIZ1	<u>LAML</u>	0.5214	0.1357	5		
PCYT1A	<u>LGG</u>	0.5194	0.1108	5		
KAT2B	<u>HNSC</u>	0.5181	0.0996	4	KAT2 proteins are differentially expressed in pancreatic cancer	[83]
SHPK	<u>BLCA</u>,STAD	0.5144	0.0583	5		
TP63	<u>BRCA</u>	0.5135	0.0707	2	rs10937405-G allele and rs4488809-G allele in TP63 might be risk-conferring factors for the development of lung cancer, especially for East Asian populations.	[84]
FN1	<u>KIRC</u>,KIRP	0.5125	0.2101	3	FN1 is a novel fusion partner of ALK in myofibroblastic tumor.	[85]
CD82	BRCA,<u>HNSC</u>	0.5116	0.0686	4	Upregulation of CD82 in gastric cancer cells resulted in the inhibition of gastric cancer migration and invasion.	[86]
EIF2AK2	<u>LAML</u>	0.5032	0.1033	4	Generally considered to have a tumor suppressor function, and some clinical data show a correlation between suppressed or inactivated EIF2AK2 and a poor prognosis for several cancers.	[87]
PHF17	<u>KIRC</u>	0.5015	0.0967	5		
MUC16	<u>LUSC</u>	0.4815	0.9759	4	MUC16-carboxy terminal mediated enrichment of cancer stem cells is partly responsible for tumorigenic, metastatic and drug-resistant properties of pancreatic cancer cells.	[88]
HUWE1	<u>KIRP</u>	0.4732	0.7558	4	Huwe1/Mcl-1 complex has a significant role in increasing the stability of Mcl-1 in breast cancer cells and	[89]

					increased resistance to apoptosis.	
RYR2	BLCA,BRCA,COADREAD,HNSC,KIRP,PRAD,STAD,UCEC	0.4727	0.5499	4	RYR2 was the most prominent change in gene expression between the epithelial and mesenchymal-like states.	[90]
SP1	BLCA,COADREAD,HNSC,KIRP,UCEC	0.3908	0.7289	4	SP1 upregulates TINCR which contributes to the oncogenic potential of gastric cancer.	[91]
PKD1	KIRP	0.3835	0.6335	4	PDK1 is critical for Lin28A- and Lin28B-mediated cancer proliferation both in vitro and in vivo.	[92]
UBR4	KIRP	0.3660	0.6418	5		
RPA1	BLCA,BRCA,KIRP,STAD,UCEC	0.3527	0.6667	5		
DNAH5	COADREAD,STAD	0.3421	0.7936	3	DNAH5 is located in 5p amplification region which is related to tumor progression in urinary bladder cancer.	[93]
LRP1B	COADREAD	0.3397	0.8203	4	LRP1B inactivation results in changes to the tumor environment that confer cancer cells an increased growth and invasive capacity.	[94]
RYR3	UCEC	0.3355	0.5951	4	SNP in microRNA which targets RYR3 affects breast cancer risk.	[95]
SYNE2	BLCA	0.3277	0.6495	5		
ANK2	BLCA,KIRP	0.3186	0.5689	4	Silencing of ANK2 expression reduced the malignant phenotype of pancreatic cancer cells.	[96]
PLEC	BLCA	0.3156	0.6551	4	Plectin involved in critical molecular process (invadopodia) for cancer cell invasion and extravasation for metastasis.	[97]
LRP2	BLCA,BRCA,COADREAD,KIRC,LGG,UCEC	0.2926	0.8180	2	Common genetic variation within the lipoprotein-related protein 2 (megalin) gene could alter both	[98]

					risk of recurrence/progression and prostate-specific cancer mortality.	
BIRC6	BLCA,KIRP	0.2911	0.5548	4	Plays a role in prostate cancer progression and treatment resistance.	[99]
FAT4	BLCA	0.2864	0.8503	2	Nonsynonymous variants rs1014867 and rs1039808 of FAT4 contribute to esophageal cancer susceptibility.	[100]
USH2A	COADREAD,KIRC	0.2826	0.5910	5		
MRE11A	BLCA,COADREAD,GBM,HNSC,LGG,LUSC,PRAD,SKCM,STAD,THCA,UCEC	0.2771	0.9920	2	MRE11A is intermediate-risk breast cancer susceptibility genes.	[101]
APOB	UCEC	0.2633	0.5607	2	Polymorphism of APOB is associated with increased risk for development of gallbladder cancer.	[102]
TAF9	BLCA,COADREAD,KIRP,STAD	0.2512	0.8256	4	TAF9 is downregulated or deleted in 98% of high-grade serous ovarian cancers.	[103]
FLG	BLCA	0.2475	0.7849	3	Amplification of flg gene correlate with loss of heterozygosity at 17p.	[104]
HMCN1	BLCA,GBM,OV,UCEC	0.2348	0.7444	5		
DLG2	BLCA,BRCA,STAD	0.2345	0.6139	4	DLG2 has decreased expression in the tumor samples compared to their matched normal tissues.	[105]
MACF1	BRCA,KIRC,UCEC	0.2237	0.5865	5		
DYNC2H1	BLCA,COADREAD,KIRC,STAD	0.2122	0.6813	2	rs716274 SNP in the region of downstream of DYNC2H1 on chromosome 11q22.3 are associated with small-cell lung cancer survival.	[106]
ROBO1	KIRP	0.2063	0.5135	4	ROBO1 expression is significantly lost in primary and metastatic tumors.	[107]
YWHAG	BLCA,BRCA,COADREAD,KIRP,STAD	0.2033	0.8134	5		

DLG3	BLCA	0.2017	0.5169	4	DLG3 is down-regulated in glioblastoma.	[108]
LGALS1	BLCA,GBM,KIRP,LUAD,PRAD,SKCM,STAD	0.2009	0.8060	4	LGALS1 is significant prognostic factor, and could be potential therapeutic targets to improve pancreatic cancer survival.	[109]
SMAD9	COADREAD,KIRP,UCEC	0.1924	0.7917	4	Identified as a novel methylation marker is identified in gastric cancer.	[110]
CSMD1	STAD	0.1889	0.5156	4	Epigenetic silencing is contributing to the reduction in CSMD1 expression in squamous cancers.	[111]
PRKACA	BLCA,COADREAD,STAD	0.1858	0.8771	5		
DNAH3	BLCA	0.1840	0.5434	5		
DLG1	BLCA,KIRP	0.1831	0.5115	4	DLG1 is mislocalized during ocular carcinogenesis and its downregulation is involved in epithelial-mesenchymal transition in mouse.	[112]
DYNC1H1	BLCA,COADREAD,KIRC,STAD,THCA,UCEC	0.1814	0.9305	4	Protein levels of Dync1h1 were decreased in primary gallbladder cancer tissues.	[113]
CELSR3	BLCA	0.1806	0.5252	5		
DMD	STAD	0.1785	0.5903	5		
TAF9B	BRCA,STAD	0.1775	0.6007	5		
GPR98	BLCA	0.1697	0.5300	5		
PXDN	BRCA,COADREAD,STAD	0.1689	0.8652	4	The adhesion-promoting effects of HO-1 (promote growth and survival of neoplastic cells) were dependent on PXDN expression.	[114]
MSLN	BRCA,COADREAD,GBM,KIRP,LUSC,SKCM,STAD	0.1446	0.8006	4	MSLN is weak diagnostic classifiers, but may be useful in a	[115]

					diagnostic biomarker panel.	
SVEP1	BRCA,STAD	0.1386	0.6208	4	SVEP1 gene expression regulation may be involved in bone-cancer-microenvironment interactions.	[116]
SMARCA2	UCEC	0.1356	0.5294	4	Loss of SMARCA2 expression is a common feature among poorly differentiated tumors in clear cell renal cell carcinomas.	[117]
ATXN1	BLCA,HNSC,KIRP,LGG,UCEC	0.1320	0.7439	5		
PFDN5	BLCA,HNSC,UCEC	0.1309	0.9897	4	Showed significantly different expressions in peripheral blood cells of patients with hepatocellular carcinoma.	[118]
CUBN	HNSC	0.1233	0.5247	5		
DLG4	BRCA,STAD,UCEC	0.1166	0.6558	4	The results suggest that DLG4 may function as a tumor suppressor in the development of human papillomaviruses-associated cancers.	[119]
RFC1	BLCA,BRCA,UCEC	0.1142	0.5276	5		
PRKACB	BRCA,COADREAD,KIRP,UCEC	0.0954	0.6707	4	PRKACB is downregulated in non-small cell lung cancer (NSCLC) tissues and that upregulation of PRKACB may be an effective way to prevent the progression of NSCLC.	[120]
PKHD1L1	KIRC	0.0951	0.5220	3	Novel fusion transcript, SBF1-PKHD1L1 is identified in T-cell large granular lymphocyte leukemia.	[121]
DST	OV	0.0943	0.5420	5		
IGSF22	STAD	0.0754	0.5703	5		
DTNBP1	BRCA	0.0728	0.5595	5		

SLC30A1	HNSC,UCEC	0.0725	0.8546	5		
PTP4A3	KIRP	0.0725	0.5539	3	Deletion of PTP4A3 reduces the tumor-initiation ability in cancer in mice.	[122]
KLF17	BLCA,BRCA,COADREAD,HNSC,STAD	0.0671	0.6000	4	KLF17 may have an oncogenic role during endometrioid endometrial cancer progression via initiating epithelial-mesenchymal transition.	[123]
DROSHA	BRCA	0.0656	0.5625	4	Altered expression of Drosha may serve as markers for disrupted miRNA biogenesis in triple negative breast cancer.	[124]
CAMK1	COADREAD	0.0645	0.5498	5		
KLF7	BLCA,KIRP,STAD	0.0644	0.7994	4	Among diffuse type gastric cancers, signet ring cell carcinoma showed lower level of methylation for KLF7 compared to poorly differentiated carcinomas.	[125]
PPAPDC1B	COADREAD	0.0615	0.5276	3	PPAPDC1B is suggested to be a common driver in the 8p11-12 amplicon in cancer.	[126]

Table References

1. Park JY, Kim SA, Chung JW, Bang S, Park SW, Paik YK, Song SY: **Proteomic analysis of pancreatic juice for the identification of biomarkers of pancreatic cancer.** *J Cancer Res Clin Oncol* 2011, **137:**1229-1238.

2. Castro M, Grau L, Puerta P, Gimenez L, Venditti J, Quadrelli S, Sanchez-Carbayo M: **Multiplexed methylation profiles of tumor suppressor genes and clinical outcome in lung cancer.** *J Transl Med* 2010, **8:**86.

3. Naba A, Clauser KR, Lamar JM, Carr SA, Hynes RO: **Extracellular matrix signatures of human mammary carcinoma identify novel metastasis promoters.** *Elife* 2014, **3:**e01308.

4. Balakrishnan A, Bleeker FE, Lamba S, Rodolfo M, Daniotti M, Scarpa A, van Tilborg AA, Leenstra S, Zanon C, Bardelli A: **Novel somatic and germline mutations in cancer candidate genes in glioblastoma, melanoma, and pancreatic carcinoma.** *Cancer Res* 2007, **67:**3545-3550.

5. Yamamoto F, Yamamoto M: **Identification of genes that exhibit changes in expression on the 8p chromosomal arm by the Systematic Multiplex RT-PCR (SM RT-PCR) and DNA microarray hybridization methods.** *Gene Expr* 2008, **14:**217-227.

6. Cunningham C, Dunlop MG, Wyllie AH, Bird CC: **Deletion mapping in colorectal cancer of a putative tumour suppressor gene in 8p22-p21.3.** *Oncogene* 1993, **8:**1391-1396.

7. Lei Y, Henderson BR, Emmanuel C, Harnett PR, deFazio A: **Inhibition of ANKRD1 sensitizes human ovarian cancer cells to endoplasmic reticulum stress-induced apoptosis.** *Oncogene* 2015, **34:**485-495.

8. Gong Y, Ren J, Liu K, Tang LM: **Tumor suppressor role of miR-133a in gastric cancer by repressing IGF1R.** *World J Gastroenterol* 2015, **21:**2949-2958.

9. Zuidervaart W, van der Velden PA, Hurks MH, van Nieuwpoort FA, Out-Luiting CJ, Singh AD, Frants RR, Jager MJ, Gruis NA: **Gene expression profiling identifies tumour markers potentially playing a role in uveal melanoma development.** *Br J Cancer* 2003, **89:**1914-1919.

10. Gokul G, Gautami B, Malathi S, Sowjanya AP, Poli UR, Jain M, Ramakrishna G, Khosla S: **DNA methylation profile at the DNMT3L promoter: a potential biomarker for cervical cancer.** *Epigenetics* 2007, **2:**80-85.

11. Fei B, Wu H: **MiR-378 inhibits progression of human gastric cancer MGC-803 cells by targeting MAPK1 in vitro.** *Oncol Res* 2012, **20:**557-564.

12. Futreal PA, Coin L, Marshall M, Down T, Hubbard T, Wooster R, Rahman N, Stratton MR: **A census of human cancer genes.** *Nat Rev Cancer* 2004, **4:**177-183.

13. Chen X, Wang Y, Zang W, Du Y, Li M, Zhao G: **miR-194 targets RBX1 gene to modulate proliferation and migration of gastric cancer cells.** *Tumour Biol* 2015, **36:**2393-2401.

14. Ding D, Lou X, Hua D, Yu W, Li L, Wang J, Gao F, Zhao N, Ren G, Li L, Lin B: **Recurrent targeted genes of hepatitis B virus in the liver cancer genomes identified by a next-generation sequencing-based approach.** *PLoS Genet* 2012, **8:**e1003065.

15. Cheng Y, Liu W, Kim ST, Sun J, Lu L, Sun J, Zheng SL, Isaacs WB, Xu J: **Evaluation of PPP2R2A as a prostate cancer susceptibility gene: a comprehensive germline and somatic study.** *Cancer Genet* 2011, **204:**375-381.

16. Huang Z, Bao S: **Ubiquitination and deubiquitination of REST and its roles in cancers.** *FEBS Lett* 2012, **586:**1602-1605.

17. Pichiorri F, Palmieri D, De Luca L, Consiglio J, You J, Rocci A, Talabere T, Piovan C, Lagana A, Cascione L, et al: **In vivo NCL targeting affects breast cancer aggressiveness through miRNA regulation.** *J Exp Med* 2013, **210:**951-968.

18. Huang Z, Wen P, Kong R, Cheng H, Zhang B, Quan C, Bian Z, Chen M, Zhang Z, Chen X, et al: **USP33 mediates Slit-Robo signaling in inhibiting colorectal cancer cell migration.** *Int J Cancer* 2015, **136:**1792-1802.

19. Johnson LG, Schwartz SM, Malkki M, Du Q, Petersdorf EW, Galloway DA, Madeleine MM: **Risk of cervical cancer associated with allergies and polymorphisms in genes in the chromosome 5 cytokine cluster.** *Cancer Epidemiol Biomarkers Prev* 2011, **20:**199-207.

20. Yamashita S, Takahashi S, McDonell N, Watanabe N, Niwa T, Hosoya K, Tsujino Y, Shirai T, Ushijima

T: **Methylation silencing of transforming growth factor-beta receptor type II in rat prostate cancers.** *Cancer Res* 2008, **68:**2112-2121.

21. Zhang Y, Li M, Wei L, Zhu L, Hu S, Wu S, Ma S, Gao Y: **Differential protein expression in perfusates from metastasized rat livers.** *Proteome Sci* 2013, **11:**37.

22. Pangon L, Mladenova D, Watkins L, Van Kralingen C, Currey N, Al-Sohaily S, Lecine P, Borg JP, Kohonen-Corish MR: **MCC inhibits beta-catenin transcriptional activity by sequestering DBC1 in the cytoplasm.** *Int J Cancer* 2015, **136:**55-64.

23. Midorikawa Y, Tsutsumi S, Taniguchi H, Ishii M, Kobune Y, Kodama T, Makuuchi M, Aburatani H: **Identification of genes associated with dedifferentiation of hepatocellular carcinoma with expression profiling analysis.** *Jpn J Cancer Res* 2002, **93:**636-643.

24. Kim Y: **Regulation of cell proliferation and migration in glioblastoma: new therapeutic approach.** *Front Oncol* 2013, **3:**53.

25. Zhu H, Yu JJ: **Gene expression patterns in the histopathological classification of epithelial ovarian cancer.** *Exp Ther Med* 2010, **1:**187-192.

26. Benusiglio PR, Pharoah PD, Smith PL, Lesueur F, Conroy D, Luben RN, Dew G, Jordan C, Dunning A, Easton DF, Ponder BA: **HapMap-based study of the 17q21 ERBB2 amplicon in susceptibility to breast cancer.** *Br J Cancer* 2006, **95:**1689-1695.

27. Heidary Arash E, Song KM, Song S, Shiban A, Attisano L: **Arhgef7 promotes activation of the Hippo pathway core kinase Lats.** *EMBO J* 2014, **33:**2997-3011.

28. Roperto S, De Tullio R, Raso C, Stifanese R, Russo V, Gaspari M, Borzacchiello G, Averna M, Paciello O, Cuda G, Roperto F: **Calpain3 is expressed in a proteolitically active form in papillomavirus-associated urothelial tumors of the urinary bladder in cattle.** *PLoS One* 2010, **5:**e10299.

29. Kamohara H, Ogawa M, Ishiko T, Sakamoto K, Baba H: **Leukemia inhibitory factor functions as a growth factor in pancreas carcinoma cells: Involvement of regulation of LIF and its receptor expression.** *Int J Oncol* 2007, **30:**977-983.

30. Xu J, Xu L, Li LT, You Q, Cha LS: **HIF1A gene Pro582Ser polymorphism and susceptibility to digestive tract cancers: a meta-analysis of case-control studies.** *Genet Mol Res* 2014, **13:**5732-5744.

31. Giubellino A, Burke TR, Jr., Bottaro DP: **Grb2 signaling in cell motility and cancer.** *Expert Opin Ther Targets* 2008, **12:**1021-1033.

32. Aguilar-Martinez E, Morrisroe C, Sharrocks AD: **The ubiquitin ligase UBE3A dampens ERK pathway signalling in HPV E6 transformed HeLa cells.** *PLoS One* 2015, **10:**e0119366.

33. Szabolcs M, Keniry M, Simpson L, Reid LJ, Koujak S, Schiff SC, Davidian G, Licata S, Gruvberger-Saal S, Murty VV, et al: **Irs2 inactivation suppresses tumor progression in Pten+/- mice.** *Am J Pathol* 2009, **174:**276-286.

34. Bomken S, Haigh S, Bown N, Carey P, Wood K, Windebank K: **Cutaneous B-lymphoblastic lymphoma with IL3/IgH translocation presenting with hypereosinophilia and acute endocarditis.** *Pediatr Blood Cancer* 2015, **62:**1055-1057.

35. Thean LF, Loi C, Ho KS, Koh PK, Eu KW, Cheah PY: **Genome-wide scan identifies a copy number variable region at 3q26 that regulates PPM1L in APC mutation-negative familial colorectal cancer patients.** *Genes Chromosomes Cancer* 2010, **49:**99-106.

36. Campa D, Butterbach K, Slager SL, Skibola CF, de Sanjose S, Benavente Y, Becker N, Foretova L, Maynadie M, Cocco P, et al: **A comprehensive study of polymorphisms in the ABCB1, ABCC2, ABCG2, NR1I2 genes and lymphoma risk.** *Int J Cancer* 2012, **131:**803-812.

37. Elhardt W, Shanmugam R, Jurkowski TP, Jeltsch A: **Somatic cancer mutations in the DNMT2 tRNA methyltransferase alter its catalytic properties.** *Biochimie* 2015, **112:**66-72.

38. Ji J, Jia S, Ji K, Jiang WG: **Wnt1 inducible signalling pathway protein-2 (WISP2/CCN5): roles and regulation in human cancers (review).** *Oncol Rep* 2014, **31:**533-539.

39. Wei JL, Fu ZX, Fang M, Zhou QY, Zhao QN, Guo JB, Lu WD, Wang H: **High expression of CASK correlates with progression and poor prognosis of colorectal cancer.** *Tumour Biol* 2014, **35:**9185-9194.

40. Han J, Lv P, Yu JL, Wu YC, Zhu X, Hong LL, Zhu WY, Yu QM, Wang XB, Li P, Ling ZQ: **Circulating methylated MINT2 promoter DNA is a potential poor prognostic factor in gastric cancer.** *Dig Dis Sci* 2014, **59:**1160-1168.

41. Gemoll T, Habermann JK, Becker S, Szymczak S, Upender MB, Bruch HP, Hellman U, Ried T, Auer G, Jornvall H, Roblick UJ: **Chromosomal aneuploidy affects the global proteome equilibrium of colorectal cancer cells.** *Anal Cell Pathol (Amst)* 2013, **36:**149-161.

42. Hu HJ, Zhang LG, Wang ZH, Guo XX: **FoxO6 inhibits cell proliferation in lung carcinoma through up-regulation of USP7.** *Mol Med Rep* 2015, **12:**575-580.

43. Thorsen K, Sorensen KD, Brems-Eskildsen AS, Modin C, Gaustadnes M, Hein AM, Kruhoffer M, Laurberg S, Borre M, Wang K, et al: **Alternative splicing in colon, bladder, and prostate cancer identified by exon array analysis.** *Mol Cell Proteomics* 2008, **7:**1214-1224.

44. Tan X, Chen M: **MYLK and MYL9 expression in non-small cell lung cancer identified by bioinformatics analysis of public expression data.** *Tumour Biol* 2014, **35:**12189-12200.

45. Huang Y, Chen J, Lu C, Han J, Wang G, Song C, Zhu S, Wang C, Li G, Kang J, Wang J: **HDAC1 and Klf4 interplay critically regulates human myeloid leukemia cell proliferation.** *Cell Death Dis* 2014, **5:**e1491.

46. Kleiber K, Strebhardt K, Martin BT: **The biological relevance of FHL2 in tumour cells and its role as a putative cancer target.** *Anticancer Res* 2007, **27:**55-61.

47. Mok SC, Kwok TT, Berkowitz RS, Barrett AJ, Tsui FW: **Overexpression of the protein tyrosine phosphatase, nonreceptor type 6 (PTPN6), in human epithelial ovarian cancer.** *Gynecol Oncol* 1995, **57:**299-303.

48. Ali K, Mahjabeen I, Sabir M, Baig RM, Zafeer M, Faheem M, Kayani MA: **Germline variations of apurinic/apyrimidinic endonuclease 1 (APEX1) detected in female breast cancer patients.** *Asian Pac J Cancer Prev* 2014, **15:**7589-7595.

49. Li W, Li X, Wang W, Li X, Tan Y, Yi M, Yang J, McCarthy JB, Xiong W, Wu M, et al: **NOR1 is an HSF1- and NRF1-regulated putative tumor suppressor inactivated by promoter hypermethylation in nasopharyngeal carcinoma.** *Carcinogenesis* 2011, **32:**1305-1314.

50. Wang Y, Xu L, Jiang L: **miR-1271 promotes non-small-cell lung cancer cell proliferation and invasion via targeting HOXA5.** *Biochem Biophys Res Commun* 2015, **458:**714-719.

51. Han X, Chen Y, Yao N, Liu H, Wang Z: **MicroRNA let-7b suppresses human gastric cancer**

malignancy by targeting ING1. *Cancer Gene Ther* 2015, **22:**122-129.

52. Geng J, Fan J, Ouyang Q, Zhang X, Zhang X, Yu J, Xu Z, Li Q, Yao X, Liu X, Zheng J: **Loss of PPM1A expression enhances invasion and the epithelial-to-mesenchymal transition in bladder cancer by activating the TGF-beta/Smad signaling pathway.** *Oncotarget* 2014, **5:**5700-5711.

53. Pallai R, Bhaskar A, Sodi V, Rice LM: **Ets1 and Elk1 transcription factors regulate cancerous inhibitor of protein phosphatase 2A expression in cervical and endometrial carcinoma cells.** *Transcription* 2012, **3:**323-335.

54. Wang JZ, Liu BG, Zhang Y: **Pin1-based diagnostic and therapeutic strategies for breast cancer.** *Pharmacol Res* 2015, **93:**28-35.

55. Abdel-Fatah TM, Middleton FK, Arora A, Agarwal D, Chen T, Moseley PM, Perry C, Doherty R, Chan S, Green AR, et al: **Untangling the ATR-CHEK1 network for prognostication, prediction and therapeutic target validation in breast cancer.** *Mol Oncol* 2015, **9:**569-585.

56. Zhang Y, Long J, Lu W, Shu XO, Cai Q, Zheng Y, Li C, Li B, Gao YT, Zheng W: **Rare coding variants and breast cancer risk: evaluation of susceptibility Loci identified in genome-wide association studies.** *Cancer Epidemiol Biomarkers Prev* 2014, **23:**622-628.

57. Elakoum R, Gauchotte G, Oussalah A, Wissler MP, Clement-Duchene C, Vignaud JM, Gueant JL, Namour F: **CARM1 and PRMT1 are dysregulated in lung cancer without hierarchical features.** *Biochimie* 2014, **97:**210-218.

58. Lin Z, Fang D: **The Roles of SIRT1 in Cancer.** *Genes Cancer* 2013, **4:**97-104.

59. Jaras M, Miller PG, Chu LP, Puram RV, Fink EC, Schneider RK, Al-Shahrour F, Pena P, Breyfogle LJ, Hartwell KA, et al: **Csnk1a1 inhibition has p53-dependent therapeutic efficacy in acute myeloid leukemia.** *J Exp Med* 2014, **211:**605-612.

60. Mitin N, Betts L, Yohe ME, Der CJ, Sondek J, Rossman KL: **Release of autoinhibition of ASEF by APC leads to CDC42 activation and tumor suppression.** *Nat Struct Mol Biol* 2007, **14:**814-823.

61. Chen C, Liang Z, Huang W, Li X, Zhou F, Hu X, Han M, Ding X, Xiang S: **Eps8 regulates cellular proliferation and migration of breast cancer.** *Int J Oncol* 2015, **46:**205-214.

62. Amamoto R, Yagi M, Song Y, Oda Y, Tsuneyoshi M, Naito S, Yokomizo A, Kuroiwa K, Tokunaga S, Kato S, et al: **Mitochondrial p32/C1QBP is highly expressed in prostate cancer and is associated with shorter prostate-specific antigen relapse time after radical prostatectomy.** *Cancer Sci* 2011, **102:**639-647.

63. Ding G, Liu F, Shen B, Feng C, Xu J, Ding Q: **The association between polymorphisms in prooxidant or antioxidant enzymes (myeloperoxidase, SOD2, and CAT) and genes and prostate cancer risk in the Chinese population of Han nationality.** *Clin Genitourin Cancer* 2012, **10:**251-255.

64. Kim H, Lee HJ, Oh Y, Choi SG, Hong SH, Kim HJ, Lee SY, Choi JW, Su Hwang D, Kim KS, et al: **The DUSP26 phosphatase activator adenylate kinase 2 regulates FADD phosphorylation and cell growth.** *Nat Commun* 2014, **5:**3351.

65. Khattar V, Thottassery JV: **Cks1: Structure, Emerging Roles and Implications in Multiple Cancers.** *J Cancer Ther* 2013, **4:**1341-1354.

66. Hsu TI, Hsu CH, Lee KH, Lin JT, Chen CS, Chang KC, Su CY, Hsiao M, Lu PJ: **MicroRNA-18a is elevated in prostate cancer and promotes tumorigenesis through suppressing STK4 in vitro and in**

vivo. *Oncogenesis* 2014, **3:**e99.

67. Khanal P, Kim G, Lim SC, Yun HJ, Lee KY, Choi HK, Choi HS: **Prolyl isomerase Pin1 negatively regulates the stability of SUV39H1 to promote tumorigenesis in breast cancer.** *FASEB J* 2013, **27:**4606-4618.

68. Cengiz B, Gunduz E, Gunduz M, Beder LB, Tamamura R, Bagci C, Yamanaka N, Shimizu K, Nagatsuka H: **Tumor-specific mutation and downregulation of ING5 detected in oral squamous cell carcinoma.** *Int J Cancer* 2010, **127:**2088-2094.

69. Menon R, Deng M, Ruenauver K, Queisser A, Peifer M, Offermann A, Boehm D, Vogel W, Scheble V, Fend F, et al: **Somatic copy number alterations by whole-exome sequencing implicates YWHAZ and PTK2 in castration-resistant prostate cancer.** *J Pathol* 2013, **231:**505-516.

70. Pasche B, Pennison MJ, Jimenez H, Wang M: **TGFBR1 and cancer susceptibility.** *Trans Am Clin Climatol Assoc* 2014, **125:**300-312.

71. Carvalho AL, Chuang A, Jiang WW, Lee J, Begum S, Poeta L, Zhao M, Jeronimo C, Henrique R, Nayak CS, et al: **Deleted in colorectal cancer is a putative conditional tumor-suppressor gene inactivated by promoter hypermethylation in head and neck squamous cell carcinoma.** *Cancer Res* 2006, **66:**9401-9407.

72. Liu L, Zhang D, Jiao JH, Wang Y, Wu JY, Huang DS: **Association between the TP53BP1 rs2602141 A/C polymorphism and cancer risk: a systematic review and meta-analysis.** *Asian Pac J Cancer Prev* 2014, **15:**2917-2922.

73. Han C, Zhao R, Liu X, Srivastava A, Gong L, Mao H, Qu M, Zhao W, Yu J, Wang QE: **DDB2 suppresses tumorigenicity by limiting the cancer stem cell population in ovarian cancer.** *Mol Cancer Res* 2014, **12:**784-794.

74. Wang NN, Li ZH, Zhao H, Tao YF, Xu LX, Lu J, Cao L, Du XJ, Sun LC, Zhao WL, et al: **Molecular targeting of the oncoprotein PLK1 in pediatric acute myeloid leukemia: RO3280, a novel PLK1 inhibitor, induces apoptosis in leukemia cells.** *Int J Mol Sci* 2015, **16:**1266-1292.

75. Wu W, Kemp BL, Proctor ML, Gazdar AF, Minna JD, Hong WK, Mao L: **Expression of DMBT1, a candidate tumor suppressor gene, is frequently lost in lung cancer.** *Cancer Res* 1999, **59:**1846-1851.

76. Cai H, Palitzsch B, Hartmann S, Stergiou N, Kunz H, Schmitt E, Westerlind U: **Antibody induction directed against the tumor-associated MUC4 glycoprotein.** *Chembiochem* 2015, **16:**959-967.

77. Hu H, Wang J, Gupta A, Shidfar A, Branstetter D, Lee O, Ivancic D, Sullivan M, Chatterton RT, Jr., Dougall WC, Khan SA: **RANKL expression in normal and malignant breast tissue responds to progesterone and is up-regulated during the luteal phase.** *Breast Cancer Res Treat* 2014, **146:**515-523.

78. Green AR, Caracappa D, Benhasouna AA, Alshareeda A, Nolan CC, Macmillan RD, Madhusudan S, Ellis IO, Rakha EA: **Biological and clinical significance of PARP1 protein expression in breast cancer.** *Breast Cancer Res Treat* 2015, **149:**353-362.

79. Wang H, Zhang L, Zhang IY, Chen X, Da Fonseca A, Wu S, Ren H, Badie S, Sadeghi S, Ouyang M, et al: **S100B promotes glioma growth through chemoattraction of myeloid-derived macrophages.** *Clin Cancer Res* 2013, **19:**3764-3775.

80. Hu C, Yi C, Hao Z, Cao S, Li H, Shao X, Zhang J, Qiao T, Fan D: **The effect of somatostatin and SSTR3 on proliferation and apoptosis of gastric cancer cells.** *Cancer Biol Ther* 2004, **3:**726-730.

81. Su W, Xu M, Chen X, Nie L, Chen N, Gong J, Zhang M, Su Z, Huang L, Zhou Q: **MiR200c targets IRS1 and suppresses prostate cancer cell growth.** *Prostate* 2015, **75:**855-862.

82. Khoronenkova SV, Dianova, II, Ternette N, Kessler BM, Parsons JL, Dianov GL: **ATM-dependent downregulation of USP7/HAUSP by PPM1G activates p53 response to DNA damage.** *Mol Cell* 2012, **45:**801-813.

83. Ansari D, Andersson R, Bauden MP, Andersson B, Connolly JB, Welinder C, Sasor A, Marko-Varga G: **Protein deep sequencing applied to biobank samples from patients with pancreatic cancer.** *J Cancer Res Clin Oncol* 2015, **141:**369-380.

84. Zhang L, Wang XF, Ma YS, Xia Q, Zhang F, Fu D, Wang YC: **Quantitative assessment of the influence of TP63 gene polymorphisms and lung cancer risk: evidence based on 93,751 subjects.** *PLoS One* 2014, **9:**e87004.

85. Ouchi K, Miyachi M, Tsuma Y, Tsuchiya K, Iehara T, Konishi E, Yanagisawa A, Hosoi H: **FN1: a novel fusion partner of ALK in an inflammatory myofibroblastic tumor.** *Pediatr Blood Cancer* 2015, **62:**909-911.

86. Zhang QH, Yao YL, Wu XY, Wu JH, Gu T, Chen L, Gu JH, Liu Y, Xu L: **Anti-miR-362-3p Inhibits Migration and Invasion of Human Gastric Cancer Cells by Its Target CD82.** *Dig Dis Sci* 2015, **60:**1967-1976.

87. Marchal JA, Lopez GJ, Peran M, Comino A, Delgado JR, Garcia-Garcia JA, Conde V, Aranda FM, Rivas C, Esteban M, Garcia MA: **The impact of PKR activation: from neurodegeneration to cancer.** *FASEB J* 2014, **28:**1965-1974.

88. Das S, Rachagani S, Torres-Gonzalez MP, Lakshmanan I, Majhi PD, Smith LM, Wagner KU, Batra SK: **Carboxyl-terminal domain of MUC16 imparts tumorigenic and metastatic functions through nuclear translocation of JAK2 to pancreatic cancer cells.** *Oncotarget* 2015, **6:**5772-5787.

89. Pervin S, Tran A, Tran L, Urman R, Braga M, Chaudhuri G, Singh R: **Reduced association of anti-apoptotic protein Mcl-1 with E3 ligase Mule increases the stability of Mcl-1 in breast cancer cells.** *Br J Cancer* 2011, **105:**428-437.

90. Davis FM, Parsonage MT, Cabot PJ, Parat MO, Thompson EW, Roberts-Thomson SJ, Monteith GR: **Assessment of gene expression of intracellular calcium channels, pumps and exchangers with epidermal growth factor-induced epithelial-mesenchymal transition in a breast cancer cell line.** *Cancer Cell Int* 2013, **13:**76.

91. Xu TP, Liu XX, Xia R, Yin L, Kong R, Chen WM, Huang MD, Shu YQ: **SP1-induced upregulation of the long noncoding RNA TINCR regulates cell proliferation and apoptosis by affecting KLF2 mRNA stability in gastric cancer.** *Oncogene* 2015, **34:**5648-5661.

92. Ma X, Li C, Sun L, Huang D, Li T, He X, Wu G, Yang Z, Zhong X, Song L, et al: **Lin28/let-7 axis regulates aerobic glycolysis and cancer progression via PDK1.** *Nat Commun* 2014, **5:**5212.

93. Zheng M, Simon R, Mirlacher M, Maurer R, Gasser T, Forster T, Diener PA, Mihatsch MJ, Sauter G, Schraml P: **TRIO amplification and abundant mRNA expression is associated with invasive tumor growth and rapid tumor cell proliferation in urinary bladder cancer.** *Am J Pathol* 2004, **165:**63-69.

94. Prazeres H, Torres J, Rodrigues F, Pinto M, Pastoriza MC, Gomes D, Cameselle-Teijeiro J, Vidal A, Martins TC, Sobrinho-Simoes M, Soares P: **Chromosomal, epigenetic and microRNA-mediated inactivation of LRP1B, a modulator of the extracellular environment of thyroid cancer cells.** *Oncogene* 2011, **30:**1302-1317.

95. Zhang L, Liu Y, Song F, Zheng H, Hu L, Lu H, Liu P, Hao X, Zhang W, Chen K: **Functional SNP in the microRNA-367 binding site in the 3'UTR of the calcium channel ryanodine receptor gene 3 (RYR3) affects breast cancer risk and calcification.** *Proc Natl Acad Sci U S A* 2011, **108:**13653-13658.

96. Chen Y, Lohr M, Jesnowski R: **Inhibition of ankyrin-B expression reduces growth and invasion of human pancreatic ductal adenocarcinoma.** *Pancreatology* 2010, **10:**586-596.

97. Sutoh Yoneyama M, Hatakeyama S, Habuchi T, Inoue T, Nakamura T, Funyu T, Wiche G, Ohyama C, Tsuboi S: **Vimentin intermediate filament and plectin provide a scaffold for invadopodia, facilitating cancer cell invasion and extravasation for metastasis.** *Eur J Cell Biol* 2014, **93:**157-169.

98. Holt SK, Karyadi DM, Kwon EM, Stanford JL, Nelson PS, Ostrander EA: **Association of megalin genetic polymorphisms with prostate cancer risk and prognosis.** *Clin Cancer Res* 2008, **14:**3823-3831.

99. Low CG, Luk IS, Lin D, Fazli L, Yang K, Xu Y, Gleave M, Gout PW, Wang Y: **BIRC6 protein, an inhibitor of apoptosis: role in survival of human prostate cancer cells.** *PLoS One* 2013, **8:**e55837.

100. Du J, Ji J, Gao Y, Xu L, Xu J, Zhu C, Gu H, Jiang J, Li H, Ma H, et al: **Nonsynonymous polymorphisms in FAT4 gene are associated with the risk of esophageal cancer in an Eastern Chinese population.** *Int J Cancer* 2013, **133:**357-361.

101. Damiola F, Pertesi M, Oliver J, Le Calvez-Kelm F, Voegele C, Young EL, Robinot N, Forey N, Durand G, Vallee MP, et al: **Rare key functional domain missense substitutions in MRE11A, RAD50, and NBN contribute to breast cancer susceptibility: results from a Breast Cancer Family Registry case-control mutation-screening study.** *Breast Cancer Res* 2014, **16:**R58.

102. Pandey SN, Srivastava A, Dixit M, Choudhuri G, Mittal B: **Haplotype analysis of signal peptide (insertion/deletion) and XbaI polymorphisms of the APOB gene in gallbladder cancer.** *Liver Int* 2007, **27:**1008-1015.

103. Ribeiro JR, Lovasco LA, Vanderhyden BC, Freiman RN: **Targeting TBP-Associated Factors in Ovarian Cancer.** *Front Oncol* 2014, **4:**45.

104. Cheickh MB, Rouanet P, Louason G, Jeanteur P, Theillet C: **An attempt to define sets of cooperating genetic alterations in human breast cancer.** *Int J Cancer* 1992, **51:**542-547.

105. Gao G, Kasperbauer JL, Tombers NM, Wang V, Mayer K, Smith DI: **A selected group of large common fragile site genes have decreased expression in oropharyngeal squamous cell carcinomas.** *Genes Chromosomes Cancer* 2014, **53:**392-401.

106. Wu C, Xu B, Yuan P, Miao X, Liu Y, Guan Y, Yu D, Xu J, Zhang T, Shen H, et al: **Genome-wide interrogation identifies YAP1 variants associated with survival of small-cell lung cancer patients.** *Cancer Res* 2010, **70:**9721-9729.

107. Parray A, Siddique HR, Kuriger JK, Mishra SK, Rhim JS, Nelson HH, Aburatani H, Konety BR, Koochekpour S, Saleem M: **ROBO1, a tumor suppressor and critical molecular barrier for localized tumor cells to acquire invasive phenotype: study in African-American and Caucasian prostate cancer models.** *Int J Cancer* 2014, **135:**2493-2506.

108. Liu Z, Niu Y, Xie M, Bu Y, Yao Z, Gao C: **Gene expression profiling analysis reveals that DLG3 is down-regulated in glioblastoma.** *J Neurooncol* 2014, **116:**465-476.

109. Chen R, Dawson DW, Pan S, Ottenhof NA, de Wilde RF, Wolfgang CL, May DH, Crispin DA, Lai LA, Lay AR, et al: **Proteins associated with pancreatic cancer survival in patients with resectable**

pancreatic ductal adenocarcinoma. *Lab Invest* 2015, **95:**43-55.

110. Kang GH, Lee S, Cho NY, Gandamihardja T, Long TI, Weisenberger DJ, Campan M, Laird PW: **DNA methylation profiles of gastric carcinoma characterized by quantitative DNA methylation analysis.** *Lab Invest* 2008, **88:**161-170.

111. Richter TM, Tong BD, Scholnick SB: **Epigenetic inactivation and aberrant transcription of CSMD1 in squamous cell carcinoma cell lines.** *Cancer Cell Int* 2005, **5:**29.

112. Vieira V, de la Houssaye G, Lacassagne E, Dufier JL, Jais JP, Beermann F, Menasche M, Abitbol M: **Differential regulation of Dlg1, Scrib, and Lgl1 expression in a transgenic mouse model of ocular cancer.** *Mol Vis* 2008, **14:**2390-2403.

113. Huang HL, Yao HS, Wang Y, Wang WJ, Hu ZQ, Jin KZ: **Proteomic identification of tumor biomarkers associated with primary gallbladder cancer.** *World J Gastroenterol* 2014, **20:**5511-5518.

114. Tauber S, Jais A, Jeitler M, Haider S, Husa J, Lindroos J, Knofler M, Mayerhofer M, Pehamberger H, Wagner O, Bilban M: **Transcriptome analysis of human cancer reveals a functional role of heme oxygenase-1 in tumor cell adhesion.** *Mol Cancer* 2010, **9:**200.

115. Kendrick ZW, Firpo MA, Repko RC, Scaife CL, Adler DG, Boucher KM, Mulvihill SJ: **Serum IGFBP2 and MSLN as diagnostic and prognostic biomarkers for pancreatic cancer.** *HPB (Oxford)* 2014, **16:**670-676.

116. Glait-Santar C, Benayahu D: **Regulation of SVEP1 gene expression by 17beta-estradiol and TNFalpha in pre-osteoblastic and mammary adenocarcinoma cells.** *J Steroid Biochem Mol Biol* 2012, **130:**36-44.

117. Xia QY, Rao Q, Cheng L, Shen Q, Shi SS, Li L, Liu B, Zhang J, Wang YF, Shi QL, et al: **Loss of BRM expression is a frequently observed event in poorly differentiated clear cell renal cell carcinoma.** *Histopathology* 2014, **64:**847-862.

118. Zhang PJ, Wei R, Wen XY, Ping L, Wang CB, Dong ZN, Deng XX, Bo W, Bin C, Tian YP: **Genes expression profiling of peripheral blood cells of patients with hepatocellular carcinoma.** *Cell Biol Int* 2012, **36:**803-809.

119. Handa K, Yugawa T, Narisawa-Saito M, Ohno S, Fujita M, Kiyono T: **E6AP-dependent degradation of DLG4/PSD95 by high-risk human papillomavirus type 18 E6 protein.** *J Virol* 2007, **81:**1379-1389.

120. Chen Y, Gao Y, Tian Y, Tian DL: **PRKACB is downregulated in non-small cell lung cancer and exogenous PRKACB inhibits proliferation and invasion of LTEP-A2 cells.** *Oncol Lett* 2013, **5:**1803-1808.

121. Izykowska K, Zawada M, Nowicka K, Grabarczyk P, Kuss AW, Weissmann R, Busemann C, Ludwig WD, Schmidt CA, Przybylski GK: **Submicroscopic genomic rearrangements change gene expression in T-cell large granular lymphocyte leukemia.** *Eur J Haematol* 2014, **93:**143-149.

122. Cramer JM, Zimmerman MW, Thompson T, Homanics GE, Lazo JS, Lagasse E: **Deletion of Ptp4a3 reduces clonogenicity and tumor-initiation ability of colitis-associated cancer cells in mice.** *Stem Cell Res* 2014, **13:**164-171.

123. Dong P, Kaneuchi M, Xiong Y, Cao L, Cai M, Liu X, Guo SW, Ju J, Jia N, Konno Y, et al: **Identification of KLF17 as a novel epithelial to mesenchymal transition inducer via direct activation of TWIST1 in endometrioid endometrial cancer.** *Carcinogenesis* 2014, **35:**760-768.

124. Avery-Kiejda KA, Braye SG, Forbes JF, Scott RJ: **The expression of Dicer and Drosha in matched normal tissues, tumours and lymph node metastases in triple negative breast cancer.** *BMC Cancer* 2014, **14:**253.

125. Yang M, Kim HS, Cho MY: **Different methylation profiles between intestinal and diffuse sporadic gastric carcinogenesis.** *Clin Res Hepatol Gastroenterol* 2014, **38:**613-620.

126. Mahmood SF, Gruel N, Nicolle R, Chapeaublanc E, Delattre O, Radvanyi F, Bernard-Pierrot I: **PPAPDC1B and WHSC1L1 are common drivers of the 8p11-12 amplicon, not only in breast tumors but also in pancreatic adenocarcinomas and lung tumors.** *Am J Pathol* 2013, **183:**1634-1644.
